# One-Dimensional (1D) Nanostructured Materials for Energy Applications

**DOI:** 10.3390/ma14102609

**Published:** 2021-05-17

**Authors:** Abniel Machín, Kenneth Fontánez, Juan C. Arango, Dayna Ortiz, Jimmy De León, Sergio Pinilla, Valeria Nicolosi, Florian I. Petrescu, Carmen Morant, Francisco Márquez

**Affiliations:** 1 Arecibo Observatory, Universidad Ana G. Méndez-Cupey Campus, San Juan, PR 00926, USA; 2 Nanomaterials Research Group, School of Natural Sciences and Technology, Universidad Ana G. Méndez-Gurabo Campus, Gurabo, PR 00778, USA; Kenneth.fontanez@gmail.com (K.F.); ortizd1@uagm.edu (D.O.); jde350@uagm.edu (J.D.L.); 3 Department of Chemistry, Purdue University, West Lafayette, IN 47907, USA; arangoj@purdue.edu; 4 CRANN and AMBER Research Centers, Trinity College Dublin, D02 Dublin, Ireland; PINILLAS@tdc.ie (S.P.); NICOLOV@tcd.ie (V.N.); 5 School of Chemistry, Trinity College Dublin, D02 Dublin, Ireland; 6 IFToMM-ARoTMM, Bucharest Polytechnic University, RO060042 Bucharest, Romania; florian.petrescu@upb.ro; 7 Department of Applied Physics, Autonomous University of Madrid and Instituto de Ciencia de Materiales Nicolas Cabrera, 28049 Madrid, Spain; c.morant@uam.es

**Keywords:** 1-D nanomaterials, nanotubes, nanofibers, nanowires, nanorods, hydrogen production, batteries, supercapacitors, photochemical cells, energy

## Abstract

At present, the world is at the peak of production of traditional fossil fuels. Much of the resources that humanity has been consuming (oil, coal, and natural gas) are coming to an end. The human being faces a future that must necessarily go through a paradigm shift, which includes a progressive movement towards increasingly less polluting and energetically viable resources. In this sense, nanotechnology has a transcendental role in this change. For decades, new materials capable of being used in energy processes have been synthesized, which undoubtedly will be the cornerstone of the future development of the planet. In this review, we report on the current progress in the synthesis and use of one-dimensional (1D) nanostructured materials (specifically nanowires, nanofibers, nanotubes, and nanorods), with compositions based on oxides, nitrides, or metals, for applications related to energy. Due to its extraordinary surface–volume relationship, tunable thermal and transport properties, and its high surface area, these 1D nanostructures have become fundamental elements for the development of energy processes. The most relevant 1D nanomaterials, their different synthesis procedures, and useful methods for assembling 1D nanostructures in functional devices will be presented. Applications in relevant topics such as optoelectronic and photochemical devices, hydrogen production, or energy storage, among others, will be discussed. The present review concludes with a forecast on the directions towards which future research could be directed on this class of nanostructured materials.

## 1. Introduction

Today, the world economy runs on fossil fuels. Several decades ago, the depletion of natural reserves of oil and natural gas was forecast, thus unlocking the full potential to develop alternative energy procedures to those based on oil. This development was also driven by the search for more ecological and less damaging processes for the environment. In the 21st century, and although it is difficult to recognize, the advances have been enormous but not enough to transform the old energy production systems. This change is an inescapable necessity, if we hope that future generations can live on the only planet we have. In this sense, one-dimensional (1D) nanostructured materials represent alternatives that have been shown to improve many energy processes, due to their extraordinary properties. In this work, we review the most relevant findings and advances in the synthesis, characterization and technological applications of 1D nanomaterials in energy generation and storage processes.

## 2. Synthesis

There are very varied synthesis procedures available for obtaining nanomaterials with applications in energy. Synthesized materials can, in turn, be assembled into higher structures with more specific applications. There are two different approaches to the synthesis of nanomaterials and the manufacture of nanostructures: (i) smaller materials can be made, reducing the scale from bulk materials, and (ii) building materials from others of smaller scale. The first method is known as "top-down" and the second as the "bottom-up" approach [1]. The top-down approach is widely used in the microelectronic industry, which pursues the miniaturization of components and circuits, with spatially arranged structures with an accuracy of only a few nanometers [2]. The most interesting feature of this method of synthesis is that the properties and some characteristics of the bulk material are maintained in the processed material, for example the composition, phase and crystalline orientation, etc. One of the most important disadvantages is the yield. From a bulk material, structured nanomaterials are obtained in a very low proportion, which represents an important economic cost and a great limitation when implementing productive processes and applications that require high yields. Top-down techniques, in turn, encompass several procedures, including ion etching [3,4,5], metal-assisted chemical etching (MACE) [6,7,8,9,10,11,12], or anodic oxidation [13,14,15,16,17,18,19,20,21,22,23,24,25,26,27,28,29,30,31,32,33].

Unlike top-down techniques, the “bottom-up” approach is based on molecular recognition and chemical self-assembly of molecules, which allows obtaining structures with sizes that can vary from a few nanometers to several microns. This approach, in turn, includes different methodologies, among which it is worth mentioning vapor-phase growth [34,35,36,37,38,39,40,41,42,43,44,45,46,47,48,49,50], liquid-phase growth [51,52], template-assisted etching [53,54,55,56,57], and electrospinning [58,59,60,61,62].

## 3. Applications

### 3.1. Photochemical Applications

Some calculations estimate that the total amount of solar radiation received over a few hours would be sufficient for the planet’s energy consumption for 1 year. For many years, systems have been developed to improve the capture processes of this solar energy. Much of the difficulty stems from the need to cover large areas in order to capture radiation, and also from the fact that solar radiation is highly dependent on the geographic region.

Recent advances in the development of more efficient semiconductors have improved the efficiency of some systems to values of around 20%. However, we are still far from values that really are a real advantage to the use of fossil fuels. Over the past few decades, tremendous strides have been made in the development and improvement of photovoltaic systems, photoelectrochemical cells, and solar hydrogen production, although we are still far from the fact that these processes may represent the first option for the planet.

#### 3.1.1. Photovoltaic Cells

Sunlight represents the most abundant renewable source of clean energy uninterruptedly available almost at any place in the globe. This resource can be utilized for various purposes which range from heating water to producing electricity through the use of photovoltaic (PV) technologies. Harvesting this incoming energy represents one of the most promising and hardly researched topics for chemists and physicists as it represents a green approach to produce energy from a source considered to be infinite. In addition, the relevant advantage of this approach over other new clean energy technologies is that sunlight can be directly converted into solar energy through solar cells. This technology offers a method to produce electrical energy in a cost-effective way avoiding the production of toxic materials as byproduct. Therefore, it stands as a pioneer within the green approaches available so far. It is expected that within the next seven years PV technologies will deliver approximately a range between 345 GW and 1081 GW and by 2050 the world energetic requirements will build up to approximately 30 TW. It is suggested that at least 20% of that necessity will be fulfilled by PV-based technologies [63].

A photovoltaic cell is a device capable to harvest solar light and further convert it into electricity. Such device is composed of semiconductor materials, among which various 1-D nanomaterials are employed within the system [64]. In brief, the main mechanism starts when the photons from sunlight are absorbed by the semiconductor, generating electrons and creating electron holes (h^+^), which are subsequently filled by other electrons resulting from the same process happening in a cascade effect in adjacent molecules. As consequence, an electron flow along the material is produced. Such an effect is known as the photovoltaic effect, and PV devices work directing these flows in a specific direction, resulting in an electrical current [65].

**Figure 1:**
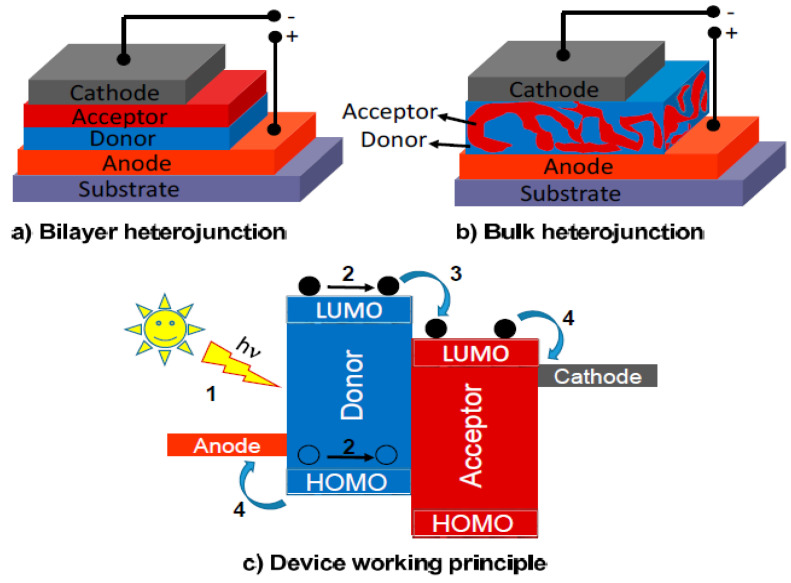
Device structures (**a**,**b**); and basic photovoltaic effect process (**c**). Reprinted with permission from reference [64].

The PV device is made of a sandwich-like stack of n-type and p-type semiconductors joined by a n-p junction where the charge separation takes place. Herein, upon light incidence, the p-type semiconductor undergoes a charge separation producing a surplus of h^+^ in the valence band. These h^+^ reach the system anode. This material is the electron donor. Simultaneously, the n-type semiconductor makes the role of electron acceptor and therefore the electrons flux flows through the material to finally reach the system cathode. A very illustrative way to visualize how this system works is thinking of the stacked layers, as presented in Figure 1 [64]. The h^+^ will migrate to the anode like an air bubble emerging from a water body, whereas the electrons being transferred at the n-p junction interphase to the acceptor can be visualized as drops of water falling. A very important aspect to take into account is that a charge separation occurs when impacting with the material and, therefore, the system depends on two main factors: the absorption efficiency of the material, which in fact is related to the capacity that has the material to absorb photons efficiently, and the optimal charge separation. Whenever charge separation occurs, these species are called excitons and describe the promotion of electrons from the valence band to the electron band of a semiconductor. Moreover, if the recombination rate increases, the cell efficiency will decrease [66,67,68].

The PV effect previously described was first reported by Alexandre-Edmond Becquerel in 1839 [69] while studying the effect of light on electrolytic cells. Nonetheless, it was only until more than 100 years later when the first modern Si solar cell was assembled by Russel Ohl [69]. Furthermore, the energy crisis of the 1970s stimulated the development of this technology.

Current solar cell devices present significant challenges for their technological improvement. Speaking strictly of PV cells (PVCs), such devices in its common configurations are brittle, and generally with a low flexibility. Therefore, their projection for use in industries such as textiles, for wearable application seems challenging. The scientific community, nonetheless, started to propose 1D materials as a means to include a flexible component to these devices that has not been considered until 2001 [70]. This development was closely followed by the implementation of 1D polymer solar cells in coaxial configurations onto optical fibers [71]. Moreover, in 2008 a testing approach was proposed instead of a coaxial path to reach the same aim [72]. From then on, different materials have been tested, looking to improve harvesting efficiencies, energy densities, and the obtention of more lightweight devices. Such approaches, materials, and results will be discussed throughout the rest of this section.

**Figure 2:**
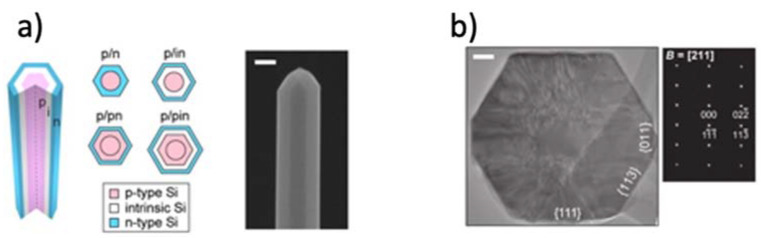
Three-dimensional schematic of a core/shell NW and cross-sectional schematics of four core/shell diode geometries, and SEM image of an as-grown, core/shell p/in Si NW, scale bar = 100 nm (**a**), and TEM image of a NW cross-section showing a core surrounded by crystalline shell, scale bar = 50 nm (**b**). Reprinted with permission from reference [73].

**Figure 3:**
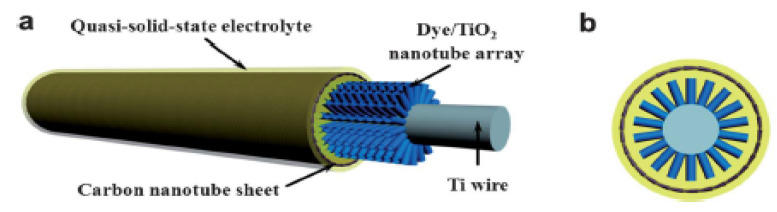
General scheme of a photovoltaic fiber with an active material sandwiched in between two electrodes, for the assembly of an FSC. Side view (**a**) and cross-sectional view (**b**) Reprinted with permission from reference [74].

The implementation of 1D materials for energy harvesting has been achieved by using coaxial structures [73] (see Figure 2). Polymorphic core/multishell NWs exhibit excellent photovoltaic properties, enhancing absorption in different regions of the solar spectrum, for the development of next-generation, ultrathin solar cells. Other examples of coaxial structures are composed of a core-shell architecture with a fiber electrode core, another electrode coating the whole system, and an active material sandwiched in-between [74] (see Figure 3).

**Figure 4:**
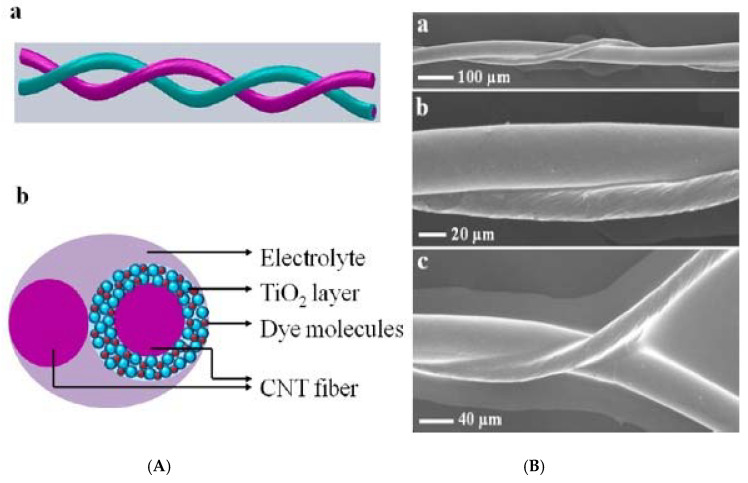
(**A**) Schematic representation of a wire-shaped FSC formed by two CNTs (one coated with TiO_2_NPs/dye; the other in its bare state) in a twisted configuration. (**B**) The SEM characterization of the system at different magnifications. Reprinted with permission from reference [83].

Typically, the semiconductor layer is composed either of TiO_2_ or ZnO nanostructures, the photoactive material (dye) and the counter electrode shell (conducting polymer or carbonaceous material). Such devices have been named as Fiber Solar Cells (FSCs), when intended for PV uses. FSCs have been proposed following two different charge transport mechanisms, photochemical and solid-state transport. For the purpose of this review, it will only be discussed photochemical transportation. In-depth solid-state transport PV materials can be found elsewhere [74]. One, is based on a photoelectrochemical transport mechanism consisting of a dye sensitized TiO_2_ nanoparticles (NPs) or nanotubes (NTs) [75,76,77], commonly referred to as dye-sensitized solar cells (DSSC). Yang et al. [78], for instance, reported an approach to produce stretchable fibers initially intended for photovoltaic technologies applied onto textile technologies, with efficiencies up to 7.13%. Herein, the fibers were initially created by winding multi walled carbon nanotubes (MWCNTs), synthesized by chemical vapor deposition (CVD) onto rubber fibers [79] following an angle α of coating ranging from 60° to 75° as the optimal values to keep the mechanical properties of the material stable, while gaining resistance thereof (0.27 to 2.4 kΩ/cm when passing from 15° to 75°). These resistances can be reduced by increasing the fiber sheath. Similarly, approaches for the fabrication of FSCs have been reported using semiconducting nanowire arrays such as CdSe [80,81], and quantum dot-sensitized ZnO nanowires [82]. Twisted structures represent the second structure used in FSCs (see Figure 4). Herein, the fiber photoanode is deposited with a semiconductor layer and further coated with a dye is wound with a fiber counter electrode [72]. Specifically, Chen et al. [83], described a system where CNT fibers dye-loaded with TiO_2_ NPs, as the working electrode and another CNT fiber used as the counter electrode were developed as FSCs. The CNT/TiO_2_ fibers were prepared by repeatedly dipping the CNT fiber into a TiO_2_ colloidal solution followed by sintering at 500 °C for 60 min. Authors attributed the high TiO_2_ NPs adsorption onto the CNT in part to the high surface area of the fiber, reaching particle thicknesses ranging from 4 to 30 µm, depending on the dipping times. This device reached an efficiency of 2.94%.

Among the most relevant favorable points to exalt from these two structures of FSCs, one can mention the high flexibility reachable by following methods as those described above. Interestingly, this flexibility allows the curves of current density as a function of voltage for the twisted architecture remains close to unchanged after bending [84].

In other modifications used to improve both the efficiency and robustness of these cells, the implementation of noble metals in junction with carbonaceous materials have been reported. For instance, MWCNTs have been dispersed and mixed with Fe_3_O_4_ or Ni NPs to reach hybrid FSCs, with efficiencies of 16.6% for the fibers coated with Fe_3_O_4_ and 11.2% for fibers with Ni NPs [85].

**Figure 5:**
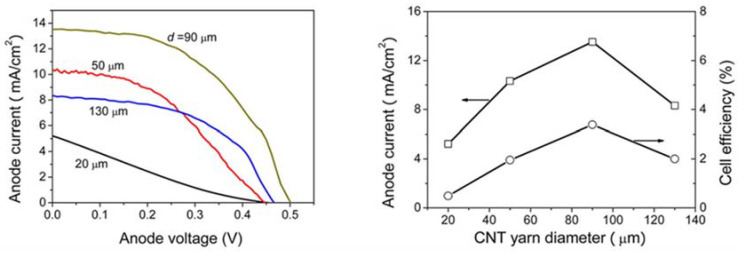
The anode current as a function of the Pt-CNT yarn diameter (**left**), and the corresponding fiber solar cells efficiency **(right**). Adapted from Zhang et al. 2012. Reproduced with permission from reference [86].

As we delve deeper into further considerations to improve the performance of PVCs limited to 1D materials, the power efficiency becomes a critical aspect to look upon, as it guarantees an acceptable output of electric power. To meet this aim, it is necessary to develop materials with good mechanical, electrical, and chemical properties [73]. For instance, the incorporation of Pt NPs to a carbonaceous material (e.g., CNTs) has been proposed as the counter electrode of titanium nanowires, with enhanced Pt-electrolyte interfacial area and a reduced charge-transfer resistance. Zhang et al. [86], reported the fabrication of TiO_2_-based dye sensitized fiber solar cells with a Pt- CNT yarns, yielding a considerable shift in current and voltage depending on the yarn diameter. The higher increase in current density (from 5.22 to 13.52 mA/cm^2^) occurred in a diameter range of 20–90 µm, with a cell efficiency change from 0.49 % to 3.38 %. However, beyond these wire dimensions, the current dropped to approximately 8 mA/cm^2^, with an efficiency of 200%. Figure 5 shows the improvement of current density as a function of yarn diameter and its corresponding cell efficiency.

The noble metal chosen as fiber electrode, must ensure proper conductivity. Among the most common materials employed, Ti [84,85,86], Al [87], and stainless-steel wires [72] stand out. Nonetheless, the implementation of materials with higher electrochemical activities such as Pt with improved methods to rough their surfaces will determine future improvements in these systems as it will enhance the further interaction of the carbonaceous materials used in these devices [73].

Electrospun nanofibers have also been applied to dye solar sensitive cells [88], specifically combining them with metallic compounds, giving rise to systems with high efficiency and stability. Chemical composition, shape, and other properties can be easily controlled by adjusting key parameters during synthesis, which has enabled the development of electrode materials for solar cells and more recently to manufacture bulk organic heterojunction solar cells and perovskite solar cells [89].

Finally, it is necessary to mention 1D perovskite NWs. Growing these materials in a low dimensional manner was first proposed as a vapor-liquid-solid growth, which enabled the growth of anisotropic perovskite NWs [90]. In this approach, a catalytic nanodroplet of a eutectic liquid alloy adsorbs the precursor in its vapor state. Furthermore, inducing a 1D anisotropic growth in the liquid-solid interphase between the crystalline material and the semiconductor [91]. Perovskites represent a material of great interest due to specific properties, such as the fact that these materials have more “softer“ crystalline lattices if compared to other semiconductors, which enables a fast crystal formation unlike other crystalline materials [92]. Moreover, various approaches can be taken to come around the production of 1D perovskites such as solution phase recrystallization growth processing [93], the vapor phase conversion method [94], direct vapor-phase growth [95], colloidal nanowire synthesis [96], space confined nanowire growth [97], nanowire growth via intermediate adducts [98], ion exchange of existing perovskite NWs [99], and NW heterostructures [100].

#### 3.1.2. Photochemical Cells

**Figure 6:**
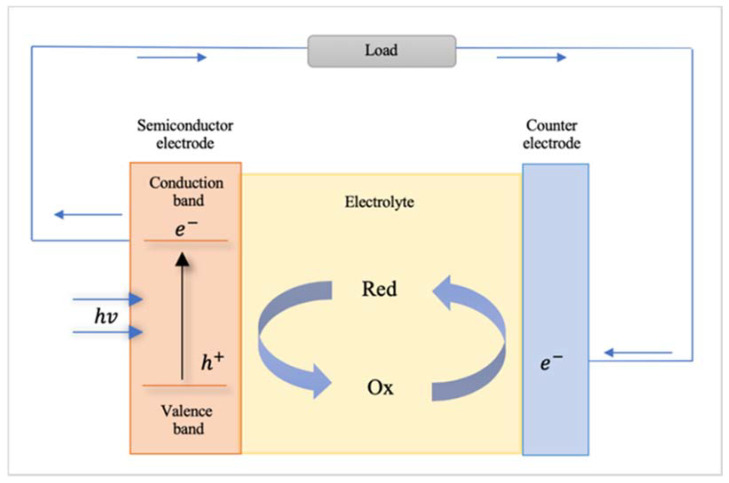
Schematic of a photoelectrochemical cell.

A photoelectrochemical cell converts light to electric power leaving no net chemical change behind [101] (see Figure 6). Photons of energy exceeding that of the band gap generate electron–hole pairs and the negative charge carriers move through the bulk of the semiconductor to the current collector and the external circuit [101]. The positive holes are driven to the surface where they are scavenged by the reduced form of the redox relay molecule (R), oxidizing it to O by the following reaction: h^+^ + R → O [101]. The oxidized form O is reduced back to R by the electrons that re-enter the cell from the external circuit [101]. In the following, some interesting examples of 1-dimensional nanomaterials used for photoelectrochemical cell applications are described.

**Figure 7:**
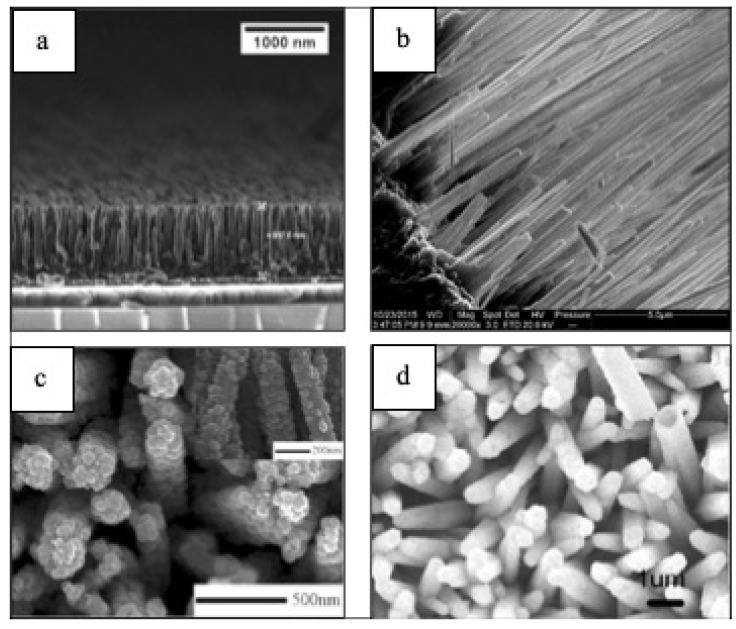
SEM characterization of (**a**) Bi:Al 21:1 photocathode [100], (**b**) CuO NWs [101], (**c**) Hydrogenated TiO_2_/ZnO heterojunction (TZ10-H) [102], and (**d**) ZnO/CdS/Au NTAs [103]. Reproduced with permission from references [102,103,104,105].

1–D morphologies (Figure 7) have shown progress when it comes to energy applications in the last five years [102,103,104,105]. For instance, it has been shown that Bi_2_O_3_/BiAl oxides nanowires (NWs) arrays (Figure 7) enhance PEC’s performance showing a hydrogen generation of up to 696 μmol cm^−2^, which corresponds to a Faradaic efficiency of 93% [102]. CuO NWs photocathodes fabricated via hydrothermal method have also shown a photocurrent of ~1.4 mA cm^-2^ at 0 V vs. RHE under AM 1.5G irradiation, which is one of the highest photocurrents based on bare CuO photocathode [103]. Hydrogenated TiO_2_/ZnO heterojunction nanorod arrays for PEC energy applications have shown photocurrent densities of nearly 2.5 mA cm^−2^, demonstrating a promising candidate for PEC cells [104]. Gold (Au) nanoparticles decorated highly ordered ZnO/CdS nanotube arrays (ZnO/CdS/Au NTAs) photoanodes exhibits a photocurrent density of 21.53 mA/cm^2^ at 1.2 V vs. Ag/AgCl and 3.45% photoconversion efficiency (PCE) among the parallel photoanodes under visible light illumination (λ > 420 nm) [105].

**Figure 8:**
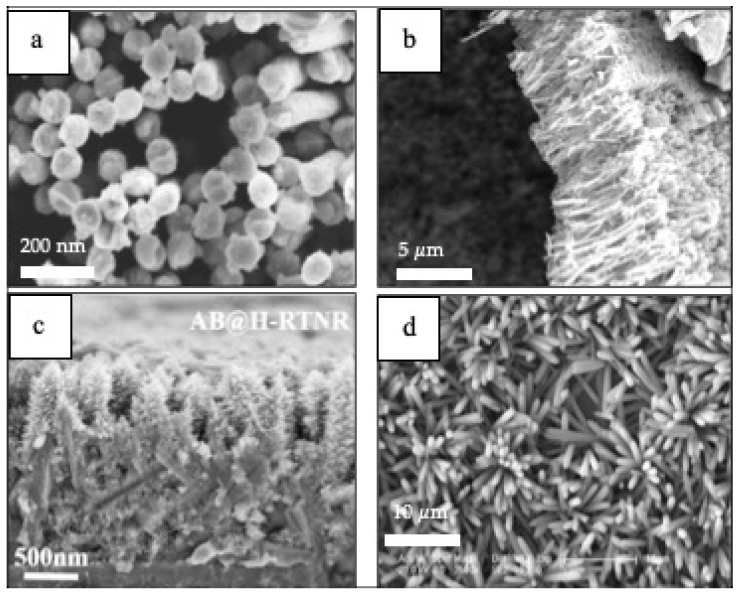
(**a**) Au–NiO_1−x_ (0 < x < 1) hybrid nanowire arrays [106], (**b**) TiO_2_ nanowires [107], (**c**) anatase-branch@hydrogenated rutile nanorod TiO_2_ [108], (**d**) ZnO nanorods and MoS_2_ flakes [109]. Reproduced with permission from references [106,107,108,109].

1–D materials (Figure 8) have also been integrated in PEC cells for sensing applications [106,107,108,109]. For instance, Au–NiO_1−x_ (0 < x < 1) hybrid NWs arrays are used as glucose sensors that exhibits an ultrahigh sensitivity of 4.061 mA cm^−2^ mM^−1^, low detection limit and a wide level of glucose concentration in the detection range of 0.005–15 mM in PEC cells [106]. In addition, TiO_2_ NWs prepared by template sol-gel synthesis are practical for a hydrazine photoelectrochemical sensor having a limit of detection (LOD) of 1.91 μM and a limit of quantification (LOQ) 8.91mM [107]. Nanorods such as high-performance anatase-branch@hydrogenated rutile-nanorod TiO_2_ have also been used for detecting chemical oxygen demand (COD) in wastewater [108]. Featuring a detection limit of 0.2 ppm and a wide linear detection range of 1.25–576 ppm [108]. A propyl gallate PEC sensor based on ZnO nanorods and MoS_2_ flakes showed a wide linear range from 1.25 10^−7^ to 1.47 10^−3^ mol L^−1^ with a detection limit as low as 1.2 10^−8^ mol L^−1^ [109].

**Figure 9:**
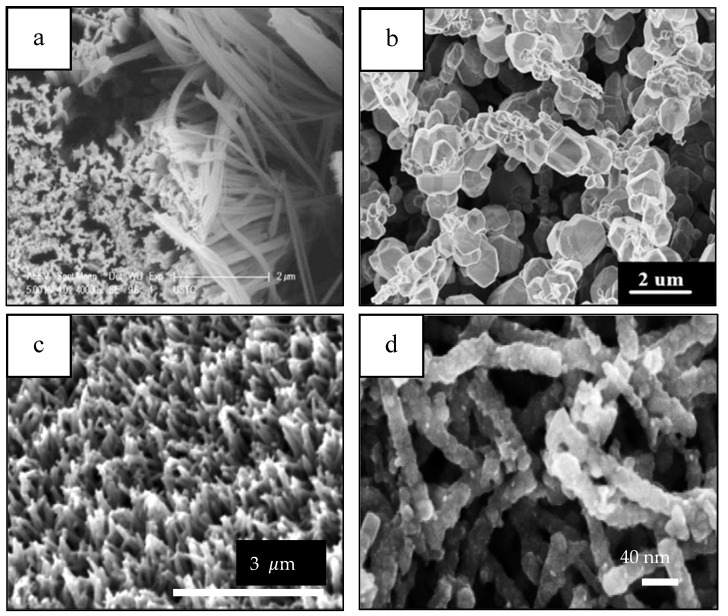
(**a**) Si nanowires [110], (**b**) C/Cu_2_O NWAs [111], (**c**) Co_3_O_4_ nanorods [112], and (**d**) Ag/AgCl @ chiral TiO_2_ nanofibers [113]. Reproduced with permission from references [110,111,112,113].

In addition, 1–D materials can also be used in PECs for other applications [110,111,112,113] (see Figure 9). A photoelectrocatalytic microbial fuel cell (photo-MFC), consisting of a palladium (Pd) NPs-modified p-type silicon (Si) NW photocathode used to degrade methyl orange (MO), and to generate electricity simultaneously exhibited a MO removal efficiency of 84.5% and maximum output power density of 0.119 W/m^2^ within 36 h [110]. A WO_3_ NFs-C/Cu_2_O NWAs visible-light response dual-photoelectrode solar-charged photoelectrochemical wastewater fuel cell (scPEWFC) was constructed for efficient hydrogen production based on the promotion of phenol oxidation at the anode [111]. The hydrogen production reaches as high as 93.08 μmol cm^-2^ by the photoelectrocatalytic oxidation of phenol (total organic carbon (TOC) removal rate reached 82.12%) of WO_3_ NFs-C/Cu_2_O NWAs under visible light irradiation for 8 h without additional bias, which is 3.02 times higher than that of pure photocatalytic water splitting [111]. A microbial photoelectrochemical cell (MPEC) with a p-type Co_3_O_4_ nanorod-arrayed photocathode for CO_2_ conversion to formic acid [112]. The yield of formic acid produced by this MPEC under visible light irradiation was 239 ± 10 μmol in 10 h and the maximum power density was 331 ± 4 mW m^−2^ under visible light [112]. In 2015, scientists developed a novel nanostructured plasmonic Ag/AgCl @ chiral TiO_2_ nanofibers (Ag and AgCl NPs supported on chiral TiO_2_ nanofibers) photoanode to treat urban wastewaters with simultaneous hydrogen production [113]. The electrolyte in the dye-sensitized solar cell (DSSC) was actual wastewater with added estrogen (17-β-ethynyl estradiol, EE2) and a heavy metal (Cu^2+^) [113]. Almost total removal of carbon (TOC), Cu^2+^, EE2, and 70% removal of total nitrogen (TN) were achieved under visible-light irradiation [113]. A relatively high solar energy conversion efficiency (PCE 3.09%) was recorded and approximately 98% of the electricity was converted to H_2_ after the consumption of dissolved oxygen (DO), Cu^2+^ and TN [113].

In conclusion, 1–D morphologies have been used in various PEC’s applications ranging from hydrogen production and sensors to even degradation of pollutants in the last five years. They have been shown to enhance performance, used for electrode stabilization, as support materials and even in conjunction with biological organisms in the case of photo-MFC. The work presented in this section proves that 1–D morphologies can adapt various roles when it comes to PEC applications, making these materials excellent candidates for multi-purpose applications given by their versatility, ease of modification, as well as their many benefits that comes from their composition characteristics.

#### 3.1.3. Hydrogen Production

It is well known that there is a necessity to find new, renewable, clean, and cost-effective sources of energy able to replace fossil fuels. In that quest, hydrogen (H_2_) have been proposed as a good candidate for the following reasons: (1) can be obtained from water; (2) is a renewable fuel; (3) can be stored as gas, liquid or solid; (4) can be transported over long distances; (5) can be converted into other forms of energy in more ways and more efficiently than any other fuel; (6) is compatible with the environment since its production, storage and end use do not produce pollutants, greenhouse gases or any other harmful effect on the environment [52,114].

In photocatalytic hydrogen production via water splitting, a catalyst with an appropriate band gap is used to absorb light and to carry out the reaction [115,116]. Usually, metal oxides such as titanium oxide (TiO_2_), copper (II) oxide (CuO), molybdenum (VI) oxide (MoO_3_), zinc oxide (ZnO), zirconium oxide (ZrO_2_), among others, are used due to their electronic structure, charge transport characteristics and light absorption properties [117]. Metal chalcogenides and metal nitrides are also widely used due to their suitable bandgaps, high catalytic currents, and electrochemical stability [118].

**Figure 10:**
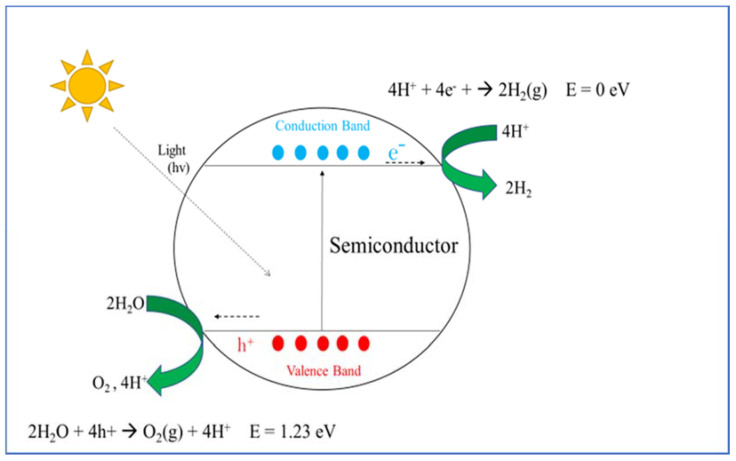
Schematic diagram of photocatalytic water splitting using a semiconductor.

In semiconductor photocatalysis, the electrons from the valence band (VB) are excited to the conduction band (CB) with light with higher energy than the respective bandgap of the semiconductor [117,119]. This migration results in the formation of an electron-pair (e^−^_cb_ / h^+^_vb_) [119], where the electrons in the CB are good reducing agents whereas the holes in VB are good oxidizing agents [116,120]. In the systems of complete water splitting, the photo-process requires the use of a semiconductor with a VB with a potential greater than the oxidation potential of water: 1.23 eV with respect to the normal hydrogen electrode (NHE, E = 0.0 V at pH = 0) [121]. Once the electrons from the VB have gained enough energy, they migrate to the CB. The holes that are formed in the VB migrate to the surface of the semiconductor where they interact with the water molecule. Water then is oxidized, releasing molecular oxygen (O_2_) and hydrogen ions (H^+^). The electrons that migrated to the CB are gained by the hydrogen ions and transformed into molecular hydrogen (H_2_) (see Figure 10). The reactions that take place are as follows:

Semiconductor + hv → h^+^_VB_ + e^−^_cb_ (1)

4h^+^_VB_ + 2H_2_O → O_2_(g) + 4H (2)

4H^+^ + 4 e^−^_cb_ → 2H_2_(g) (3)

**Figure 11:**
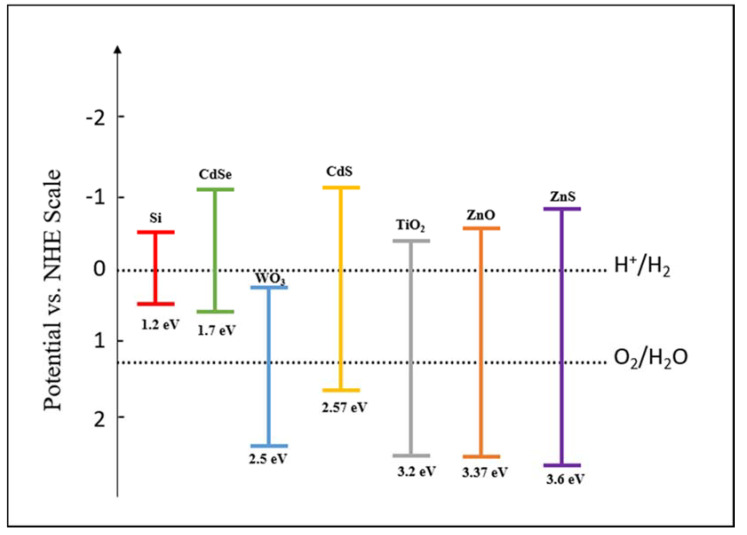
Bandgap potential diagram of semiconductors at normal hydrogen electrode.

As a result of the loss of energy due to the existence of barriers in the transference of electrons and the overpotential for the release of hydrogen and oxygen, the value of 1.23 eV increases to 1.7–1.9 eV [119,122]. This means that the photocatalytic conversion of the solar energy should occur, more satisfactorily, in systems with semiconductors with bandgaps over the range of 1.7–1.9 eV. There are multiple semiconductors that fulfill this requirement and the most common are presented in Figure 11. Even though a lot of these semiconductors have a suitable bandgap, some of them have a bandgap edge that does not favor the decomposition of water (WO_3_), others are unstable (CdS, CdSe) or have bandgaps over 3.0 eV (TiO_2_, ZnO) that do not allow the use of visible light [119,123].

During the last decades, multiple efforts have been made to find suitable materials that fit all the requirements mentioned previously. Among these, one-dimensional (1D) nanostructured materials such as nanowires, nanorods, nanotubes and nanofibers have been used as photocatalysts to produce hydrogen via water splitting. Some of the advantages that these materials exhibit are high surface area, surface–volume relationship, and tunable thermal and transport properties [124,125].

Nanowires are typically 1000 times, or more, larger than their diameter, and because of this massive difference in length they have high surface area and, make them very sensitive to changes in surface chemistry [126]. This property is not seen in bulk materials and this gives them unique advantages to be used as catalysts in some reactions such as water splitting. There are multiple synthesis processes to obtain nanowires and depending on several factors (precursors, temperature, pressure, among others), their length, shape, and catalytic properties could change.

**Figure 12:**
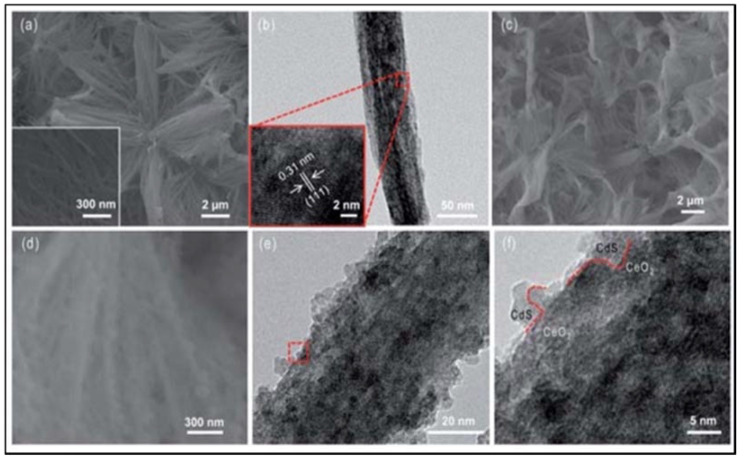
SEM (**a**) and TEM (**b**) images of the CeOx nanowires. The SEM and TEM images of the CdS/CeOx nanowires are presented in (**c**–**f**). The inset in (**b**) corresponds to (**a**) HRTEM image and SAED spectrum of the CeOx nanowires. Reproduced with permission from reference [128].

For the last decade a lot of efforts have been made to obtain nanowires suitable to be used as photocatalysts for the production of hydrogen via water splitting. Li and group [127] synthesized cerium oxide (CeO_2_) nanowires on a copper (Cu) substrate via an electrochemical deposition without templates. CeO_2_ is part of the rare earth oxides and have gained attention in recent years due to its relatively small band gap of 3.2 eV and strong redox capability [127]. To enhance the light-harvesting capability of the nanostructure, and to be able to use visible light, the authors incorporated cadmium sulfide (CdS) nanoparticles onto the surface of the nanowires. CdS is a metal chalcogenide and is known to act as a visible-light photocatalyst, photosensitizer and to have band-edges suitable for water splitting [127]. The SEM and TEM images of the different catalysts are shown in Figure 12. The selected area electron diffraction (SAED) and TEM of the CeOx nanowires confirmed that the wires were polycrystalline with a lattice fringe of 0.31 nm (inset of Figure 12b). The images also confirmed the incorporation of CdS nanoparticles on the surface of the nanowires. The CdS/CeOx heterostructured nanowires exhibited substantially higher photocatalytic activity for hydrogen production than the pristine CeOx nanowires, showing hydrogen production of 1290.5 µmol g^−1^ h^−1^ under white light irradiation and 473.6 µmol g^−1^ h^−1^ under visible-light irradiation.

**Figure 13:**
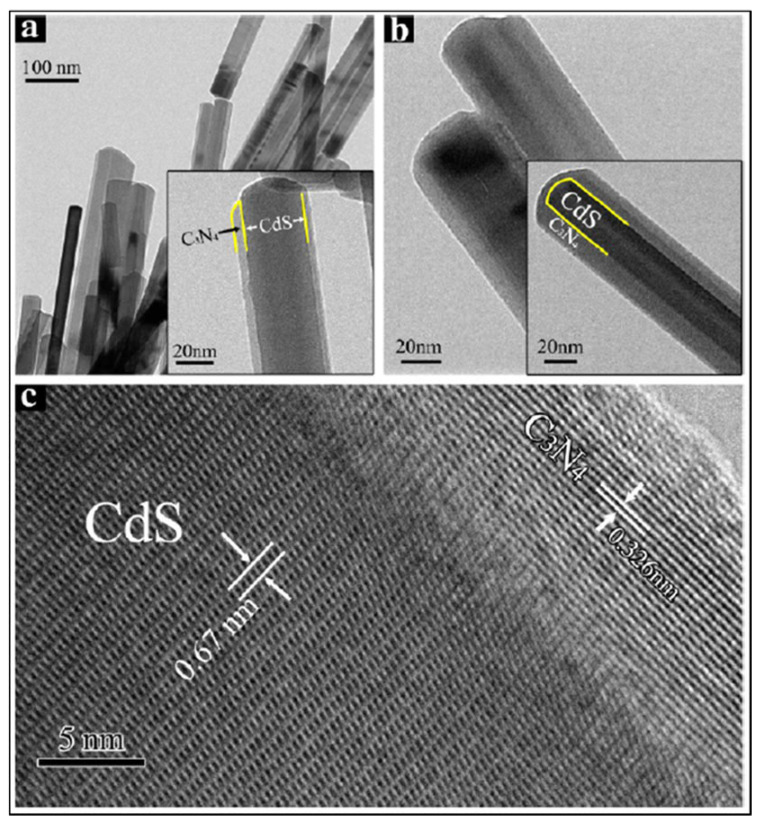
TEM images of the CdS/g-C_3_N_4_ nanowires with 2 (**a**) and 4 (**b**) wt.% of g-C_3_N_4_. The HRTEM image of CdS/g-C_3_N_4_ with 4 wt% g-C_3_N_4_ is presented in (**c**). Reproduced with permission from reference [128].

Other researchers [128,129] have employed solvothermal approaches to synthesize nanowires. Solvothermal methods consist in placing reactants into an autoclave filled with an organic compound to carry out the reaction under high temperature and pressure conditions [130]. Zhang and coworkers [128] synthesized CdS nanowires by solvothermal method adding cadmium (II) nitrate, thiourea and ethylenediamine in a Teflon-line autoclave. One of the main challenges of using semiconductors such as CdS is the low efficiency in the hydrogen production due to fast recombination of photoexcited charge carriers and the photo-corrosion of the material [128]. To deal with these limitations, graphitic carbon nitride (g-C_3_N_4_) was incorporated to the as-synthesized CdS nanowires by a two-step self-assembly procedure. The first step consisted in grounding g-C_3_N_4_ to a fine powder and exfoliate them into thin sheets to create a homogeneous suspension, followed by the addition of a desired amount of CdS nanowires. The authors synthesized CdS nanowires with different amounts of g-C_3_N_4_ (0, 0.5, 1, 2, 3, and 4 wt %) (see Figure 13). The HRTEM (Figure 13c) showed d-spacing of 0.67 nm corresponding to CdS, whereas the lattice fringes with 0.326 nm corresponded to C_3_N_4_, ascribed to the (002) interlayer–stacking distance of g-C_3_N_4_. To evaluate the hydrogen production, the authors also incorporated 0.6 wt% of platinum (Pt) and reported that the catalyst with the higher hydrogen production via water splitting was the catalyst containing 2 wt % of g-C_3_N_4_.

**Figure 14:**
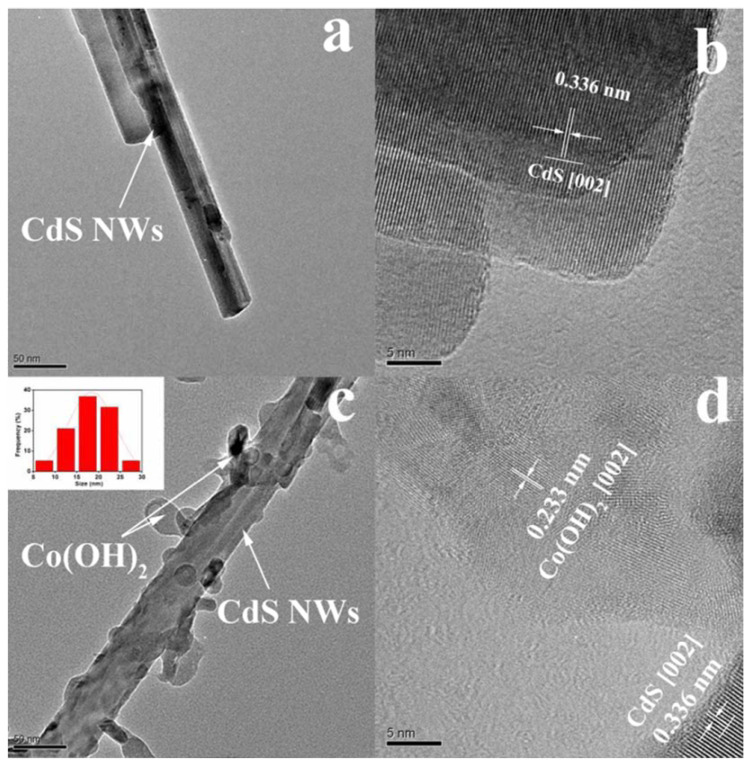
TEM and HRTEM images of (**a**,**b**) for CdS NWs. (**c**) (insert: the particle distribution of Co(OH)_2_ clusters on CdS NWs) and (**d**) Co(OH)_2_/CdS NWs with a 6.5 mol% of Co(OH)_2_. Reproduced with permission from reference [129].

Other authors [129] enhanced the H_2_ production, by adding different amounts of cobalt (II) hydroxide (Co(OH)_2_; (0, 0.5, 4.8, 6.5, and 9.1 mol%) to the as-synthesized CdS nanowires by a precipitation method. The incorporation of the Co(OH)_2_ effectively accelerated the charge separation and transfer in photocatalytic reactions, leading to an enhanced H_2_ production rate. Figure 14 shows the TEM images of the Co(OH)_2_/CdS NWs and it can be observed that many pendant-like Co(OH)_2_ clusters with diameter ca. 10–30 nm were deposited on the surface of CdS NWs with diameters of ca. 30–40 nm (Figure 14c). HRTEM showed high crystallinity for the CdS NWs and low crystallinity of Co(OH)_2_ (Figure 14d). Among the different catalysts, the one with 6.5 mol% of Co(OH)_2_ obtained the highest hydrogen production, 14.43 mmol g^−1^ h^−1^ at a wavelength λ ≥ 420 nm, which was 206 times higher than the pristine CdS NWs and three times higher than the 1wt% Pt/CdS NWs they used as a benchmark.

**Figure 15:**
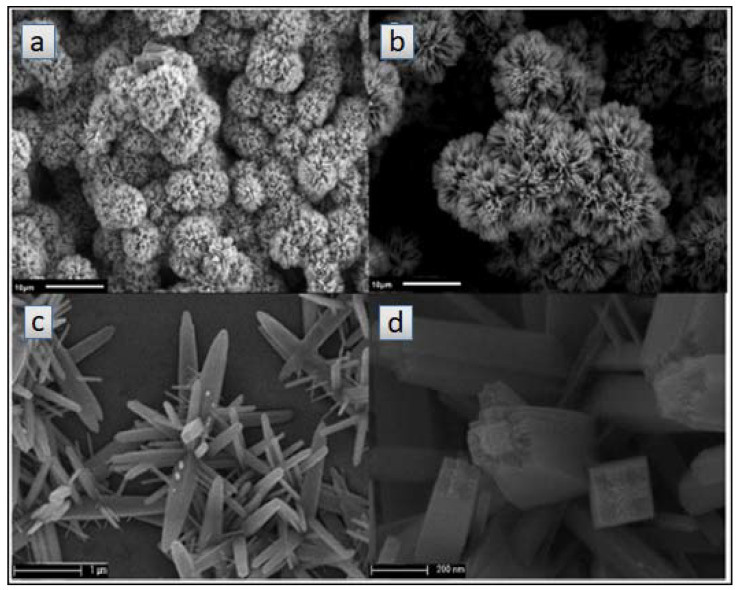
SEM images of the TiO_2_ NWs at different magnifications: 2500× (**a**). 5000× (**b**), 33,000× (**c**), and 50,000× (**d**). Reproduced with permission from reference [131].

Machín et al. [131] used a hydrothermal approach [130] to synthesize TiO_2_ nanowires. The synthesis consisted of a mixture of concentrated hydrochloric acid (HCl), titanium tetrachloride (TiCl_4_) and water placed in a Teflon-stainless stainless-steel autoclave. Figure 15 shows SEM images of the TiO_2_ NWs at different magnifications. TiO_2_ NWs consisted of homogeneous and highly branched structures. At higher magnification (Figure 15c,d), it consists of a randomly arranged material with different domains and sizes. To enhance the photocatalytic activity of the nanowires, the authors incorporated different amounts (1, 3, 5, and 10 wt.%) of gold nanoparticles (Au NPs) by a chemical reduction method using chloroauric acid (HAuCl_4_) and sodium borohydride (NaBH_4_). The incorporation of Au NPs reduced the fast recombination of the photogenerated charge carriers, enabling the use of visible light. The catalyst with the highest hydrogen production (1436 µmol g^−1^ h^−1^) was the one with an Au loading of 10 wt.%. The enhancement in the hydrogen production was 11.5 times higher than that reported by bare TiO_2_ NWs catalyst (125 μmol g^−1^ h^−1^).

**Figure 16:**
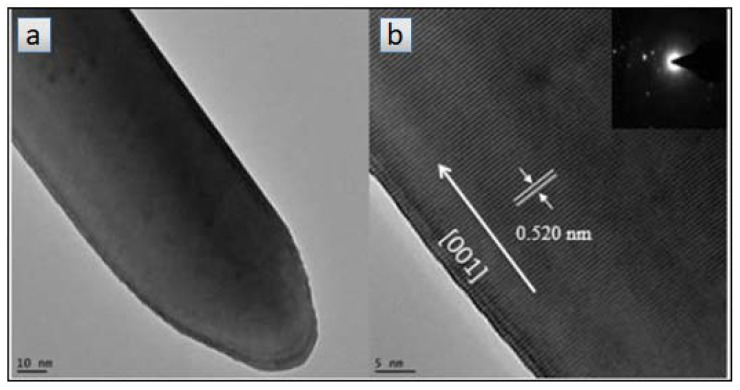
HRTEM images of the ZnO NWs at different magnifications. The inset corresponds to the SAED pattern of the ZnO NWs (D). Reproduced with permission from reference [132].

Another method to synthesize nanowires is by thermal decomposition. This method involves a single-step process, it is inexpensive, environmentally friendly, and provides high quality in terms of morphology, size, and particle-size distribution [130]. Machín and coworkers [132] synthesized zinc oxide nanowires (ZnO NWs) by a simple thermal decomposition method. The synthesis consisted in the thermal treatment of 0.5 g of zinc acetate dehydrate (Zn(CH_3_COO)_2_ 2H_2_O) in an alumina crucible at 300 °C for three hours. The HRTEM images of the synthesized ZnO NWs are shown in Figure 16. The lattice spacing of the ZnO NWs was ca. 0.52 nm between adjacent planes, revealing that preferential growth of the ZnO NWs was oriented on the c-axis.

One of the main disadvantages of ZnO when compared with TiO_2_ is that photocorrosion often occurs, decreasing the catalytic activity [132]. As occurs with TiO_2_, the high bandgap (3.37 eV) of the semiconductor does not allow the use of visible light to produce hydrogen via water splitting. To deal with these limitations, the authors incorporated different amounts of Au NPs (1–10 wt.%.) by a chemical reduction method using NaBH_4_ as a reducing agent. The highest hydrogen production was 853 μmol g^−1^ h^−1^, obtained with an Au loading of 10 wt.% at a wavelength of 400 nm.

Other 1D nanostructured materials that have gained a lot of attention for the last decade are nanorods. Nanorods have typically lengths of 10 to 20 nm and have the advantage that can be made of metals, nonmetals, or mixed compounds [133]. The requirements for their production are more flexible than those for nanotubes and nanowires. 

**Figure 17:**
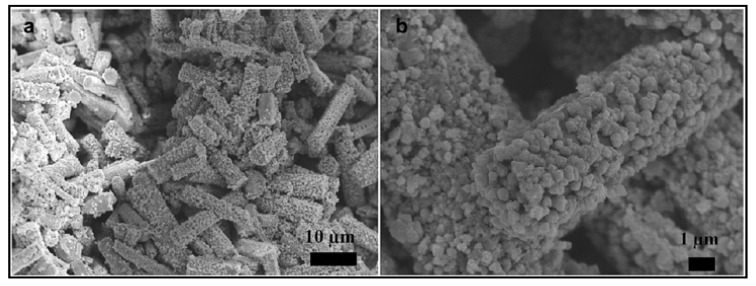
FE-SEM images of the CuO/ZnO nanorods at different magnifications. Reproduced with permission from reference [134].

Liu and group [134] synthesized ZnO nanorods by a hydrothermal method using Zn(CH_3_COO)_2_, hexamethylenetetramine (HMTA, C_6_H_12_N_4_), sodium hydroxide and HCl as precursors and heating the mixture at 95 °C for 6 hours. As mentioned before, some of the disadvantages of ZnO as catalyst for hydrogen production include the low photocatalytic efficiency, high bandgap (3.37 eV), recombination of photogenerated electrons and holes and photocorrosion [131,134]. To overcome these difficulties, the authors synthesized a hierarchically structure with a multi-scale organization by adding copper oxide (CuO) to the as-synthesized ZnO nanorods. The advantages of these nanostructures include: (1) enlarging the light utilization rate by shifting the light absorption to the visible range, (2) maximizing the specific surface area for mass transfer and reactants access by creating porous interior spaces, and (3) retarding the recombination of photogenerated electrons and holes. The addition of CuO consisted in mixing the ZnO nanorods in a solution that contained copper (II) sulfate pentahydrate (CuSO_4_ 5H_2_O), and sodium chloride (NaCl). Figure 17 shows the FE-SEM images of CuO/ZnO rods at different magnifications, showing that only partial surface of the ZnO rods were covered by CuO nanoparticles and that there is enough space between CuO nanoparticles. The bare ZnO nanorods had a negligible H_2_ production but an estimated amount of 1700 µmol g^−1^ h^−1^ was obtained with the CuO/ZnO catalyst. This enhancement was justified as due to the efficient use of the irradiation, the specific surface area increased, and the recombination of the holes and photo-excited electrons decreased.

**Figure 18:**
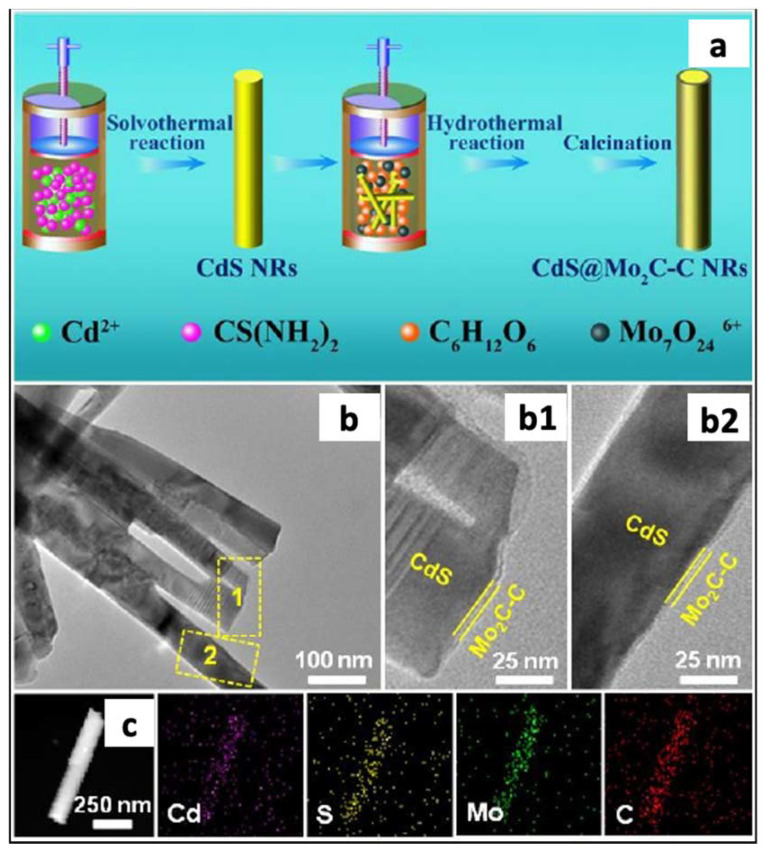
(**a**) Scheme illustration of the preparation process for CdS@Mo_2_C-C core-shell nanorods. (**b**) TEM images of CdS@1Mo_2_C-C core-shell nanorods; (**b1**,**b2**) the enlarged view of the circles areas in b; and (**c**) STEM-EDX elemental mapping for CdS@1Mo_2_C-C core-shell nanorods. Reproduced with permission from reference [135].

Other researchers [135] synthesized CdS nanorods by mixing cadmium (II) nitrate (Cd(NO_3_)_2_), thiourea, and ethylenediamine at 160 °C. The efficiency of CdS nanorods as photocatalyst for hydrogen production was affected by the fast recombination of the photogenerated electron-hole pairs and the photoinstability due to its high tendency to be oxidized by the photogenerated holes. To overcome these limitations, these catalysts were modified by adding molybdenum carbide (Mo_2_C). Mo_2_C has high electric conductivity and high catalytic properties compared to other Pt-group metal carbides. Figure 18a shows the scheme of the preparation process as well as TEM images of the as-synthesized CdS@1Mo_2_C-C core shells nanorods. The TEM images (Figure 18b) showed that, when compared to pristine CdS, some nanolayers are coated on the surface of the CdS nanorods to form the core-shell hybrid. The elemental mapping images (Figure 18c) identified the presence of Cd, S, Mo, and C elements.

**Figure 19:**
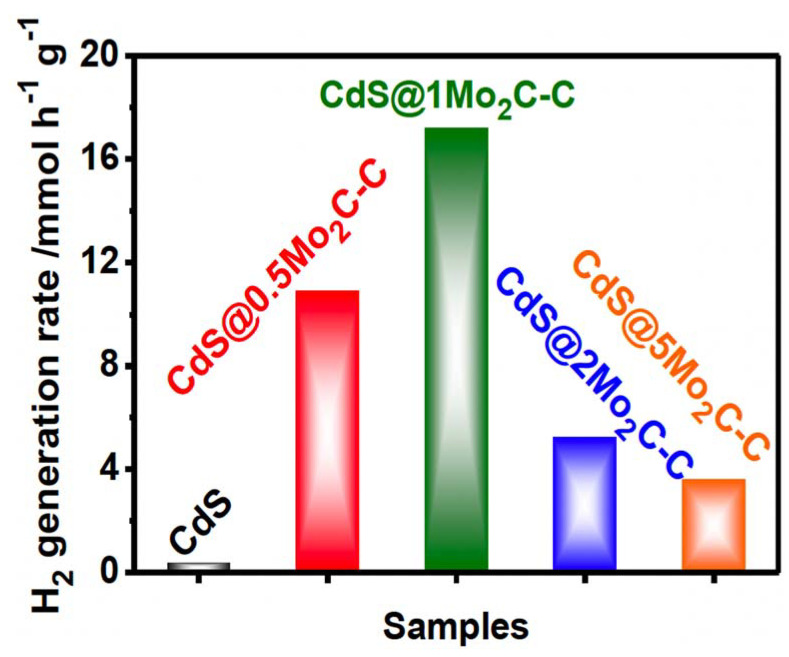
Photocatalytic hydrogen generation activities on CdS and CdS@xMo_2_C-C (x = 0.5, 1, 2, and 5, where x refers to the theoretical weight percent value of Mo_2_C). Reproduced with permission from reference [135].

Other catalysts containing different amounts of Mo_2_C-C were prepared by modifying the amounts of (NH_4_)_6_Mo_7_O_24_·4H_2_O and C_6_H_12_O_6_. The results (see Figure 19) showed that the pure CdS nanorods produced 0.41 mmol g^−1^ h^−1^ and the CdS@Mo_2_C-C ca. 17.24 mmol g^−1^ h^−1^. This huge difference in hydrogen production was ascribed to the unique one-dimensional nanostructure, the strong interface interaction between the core and shell materials, as well as the broadened visible-light absorption range. Furthermore, the presence of C layers in the core-shell nanorods can facilitate the transferring of the photogenerated holes to the outer shell of Mo_2_C-C, and thus protect the inner CdS from photocorrosion. 

**Figure 20:**
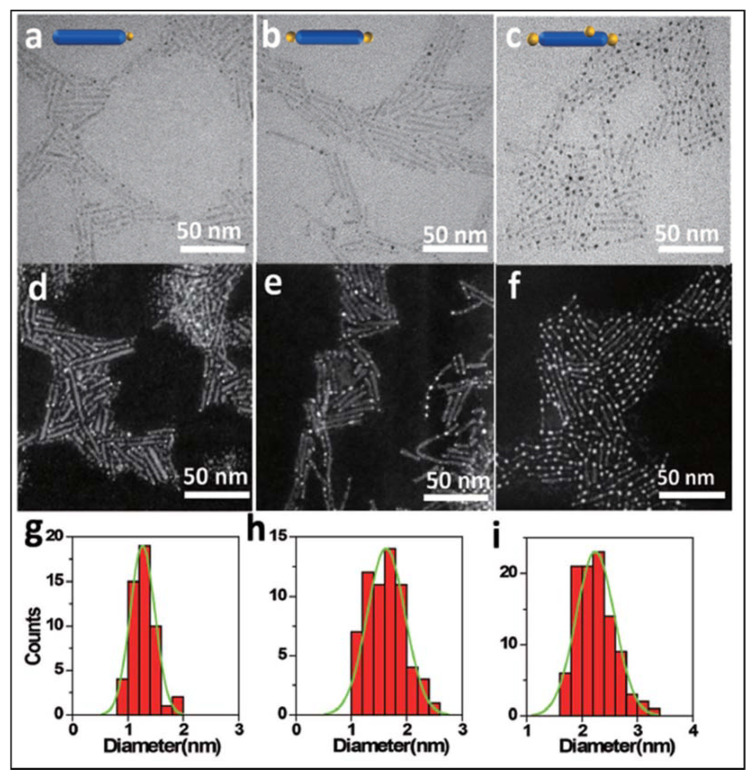
(**a**–**c**) TEM images of Au–ZnSe hybrid nanorods with Au tips of variable size. (**d**–**f**) STEM images corresponding to (**a**–**c**). (**g**–**i**) Particle size histograms corresponding to (**a**–**c**). Reproduced with permission from reference [136].

Another technique that has been used to synthesize nanorods is heat-up method. Chen and coworkers [136] used this method and synthesized colloidal gold (Au)–ZnSe hybrid nanorods. These structures not only have properties of individual components but also manifest synergistic behavior from the interaction which make them suitable for multiple purposes such as hydrogen generation, CO_2_ reduction, photodynamic therapy, among others. The synthesis of the ZnSe nanorods consisted in mixing Zn(CH_3_COO)_2_, selenium, 1-dodecanethiol (DDT) and oleylamine (OLA), and then the temperature of the mixture was raised to 260 °C. The growth of Au tips on ZnSe nanorods was made by mixing ZnSe with toluene, gold (III) chloride (AuCl_3_), and dodecylamine (DDA). TEM and high-angle annular dark-field-STEM (HAADF-STEM) images of the Au–ZnSe catalysts are shown in Figure 20. It can be seen that very small gold tips (dark spots in Figure 20a, bright spots in Figure 20d) with diameters of ca. 1.3 ± 0.2 nm grew onto one of the two apices of the ZnSe nanorods. Higher the gold concentration, higher the number of Au tips deposited on the ZnSe nanorods (Figure 20b,c,e,f).

The hydrogen production obtained with the Au–ZnSe hybrid nanorods was 437.8 µmol g^−1^ h^−1^ whereas the bare ZnSe obtained 49.8 µmol g^−1^ h^−1^. This result was justified by the enhanced charge separations [136].

Sol–gel is another popular method to synthesize nanorods [130]. This approach consists in the hydrolysis of the metal precursor, followed by a condensation step. After stirring, a gel is formed, that is subsequently submitted to a drying process. producing xerogels (evaporative drying) or aerogels (supercritical drying). TiO_2_ nanorods were synthesized by a sol-gel method [137] and, after that, a solvothermal procedure was used to incorporate different amounts of bismuth sulfide (Bi_2_S_3_) onto the surface of the nanorods. TiO_2_ has been extensively used due its high stability, low cost, availability and nontoxicity but is not appropriate for hydrogen production by water splitting, due to the fast recombination of the electron-hole pair [137]. By incorporating Bi_2_S_3_, the efficiency of electron-hole separation increases, as well as the interfacial charge transfer rate of the photogenerated charge carriers, leading eventually to a higher efficiency. The synthesis of the TiO_2_ nanorods consisted in mixing water, 1-butanol, nitric acid and of titanium (IV) butoxide. Different amounts of Bi_2_S_3_ (3, 6, 9 wt.%) were subsequently incorporated and the catalytic activity was tested. The maximum hydrogen production was 2460 µmol g^−1^ h^−1^ under UV light, being four times greater than bare TiO_2_ (564 µmol g^−1^ h^−1^).

**Figure 21:**
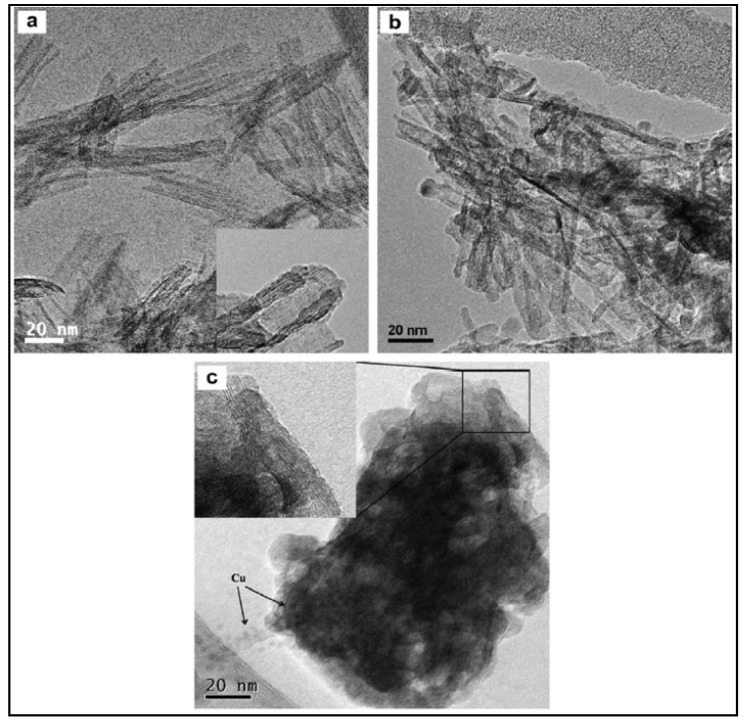
HRTEM images of photocatalysts: TNT showing a multilayered nanotubular structure (**a**); CuO incorporated onto TNT by adsorption–calcination (**b**); and CuO incorporated by wet-impregation (**c**). The inset in c shows a higher magnification of the aggregation of the nanotubes. Reproduced with permission from reference [139].

Other 1D materials that are often used for the production of hydrogen are nanotubes [138]. Xu et al. [139] incorporated CuO to TiO_2_ nanotubes to be used for hydrogen production by water splitting. TiO_2_ nanotubes (TNT) have advantages over other structures due to their large specific surface area, mesoporous structure, high aspect ratio, and efficient electron conductivity, but little is known of their capacity as photocatalyst for hydrogen production. The synthesis of the TNT was made by hydrothermal method. The addition of CuO onto the as-synthesized TNT consisted in two different methods: adsorption–calcination (A-C) and wet impregnation (WI). CuO is a cost-effective material that possesses a good photocatalytic activity for water reduction, since copper compounds facilitate the charge separation and provide reduction sites for hydrogen formation. The morphology of the catalysts was studied by HRTEM (see Figure 21). The TNT has a multilayered nanotubular structure (Figure 21a), and most of the nanotubes were open at both ends. With copper incorporation (Figure 21b,c), the sample still maintained a similar tubular structure while some aggregation of nanotubes occurred (Figure 21c).

This research reported negligible hydrogen production from the bare TNT but a significant increase with the CuO-TiO_2_ catalysts. Chen et al. [138] synthesized TNTs and enwrapped them onto CdS nanoparticles. CdS nanocrystals, upon excitation with visible light, inject electrons into the TNTs and the photoexcited holes stay in the VB of CdS to react with the sacrificial agents (Na_2_SO_3_, Na_2_S).

**Figure 22:**
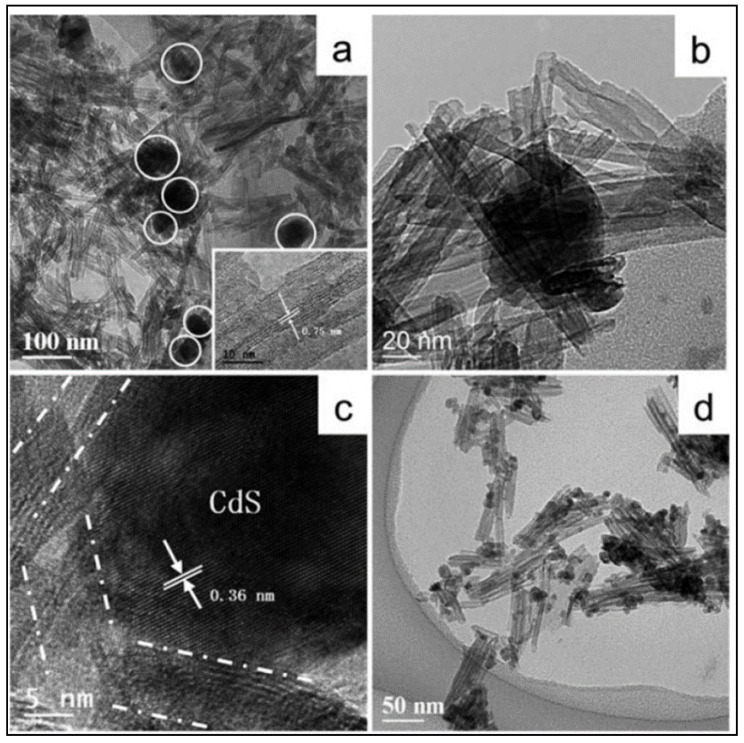
TEM images of various samples: (**a**,**b**) CdS/TNTs; (**c**) the multipoint contacts between the CdS nanoparticles and TNTs in CdS/TNTs; (**d**) CdS@TNTs. Reproduced with permission from reference [140].

The morphology and structure of the catalysts is shown in Figure 22. As it can be seen, TNTs were uniformly distributed with an average outer diameter of ca. 10 nm and average inner diameter of 4 nm. The interlayer spacing of the multilayer nanotubes was about 0.75 nm (see Figure 22a,b). The lattice fringes of the CdS monocrystalline with spacing of 0.36 nm is presented in Figure 22c.

**Figure 23:**
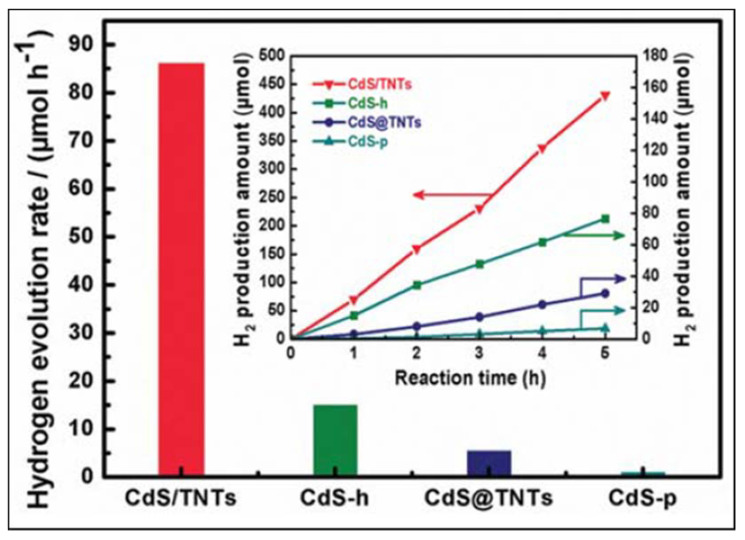
Hydrogen evolution rates over CdS/TNTs, CdS@TNTs, CdS-h and CdS-p. The inset shows their time courses of hydrogen evolution. Reproduced with permission from reference [140].

For comparative reasons, 2 wt.% of Pt was added to the as-synthesized CdS/TNTs. The hydrogen production was measured under irradiation at 430 nm. The highest hydrogen production was 353.4 µmol h^−1^ and was obtained with the catalyst with a 0.05 Cd to Ti molar ratio and 2 wt.% Pt (see Figure 23).

**Figure 24:**
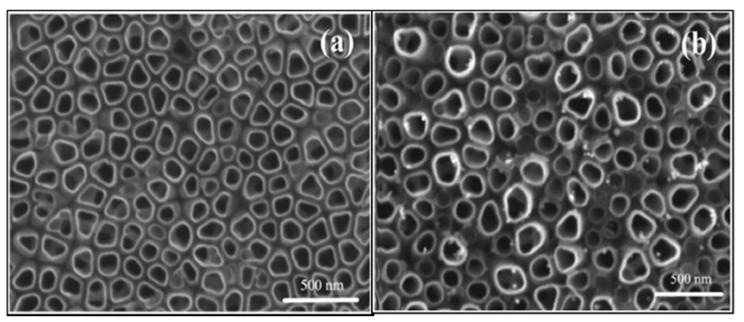
SEM images: (**a**) pure TiO_2_ NTs, (**b**)Fe doped and Ag NPs loaded on TiO_2_ NT. Reproduced with permission from reference [141].

TNTs were also synthesized by an electrochemical method [141]. TNTs were fabricated by potentiostatic anodization using Ti sheet as the working electrode and highly pure graphite as counter electrode. Subsequently, TNTs were doped with Fe^3+^, and different amounts Ag NPs. These chemical modifications allow absorption improvements in the visible light region due to the effect of localized surface plasmon resonance (LSPR) produced by the collective oscillation of the surface electrons. Ag NPs enhance the efficiency of electron-hole separation by forming a Schottky barrier at the Ag/TiO_2_, improving the photocatalytic activity. The SEM images of the pure TNTs and Fe-doped and Ag NPs loaded on TNTs are shown in Figure 24. It can be observed that highly ordered and vertically aligned TNTs were obtained with an average pore diameter of 60 nm. Ag NPs were bound uniformly both outside and within the TNTs, without affecting the ordered array structure of the NTs.

**Figure 25:**
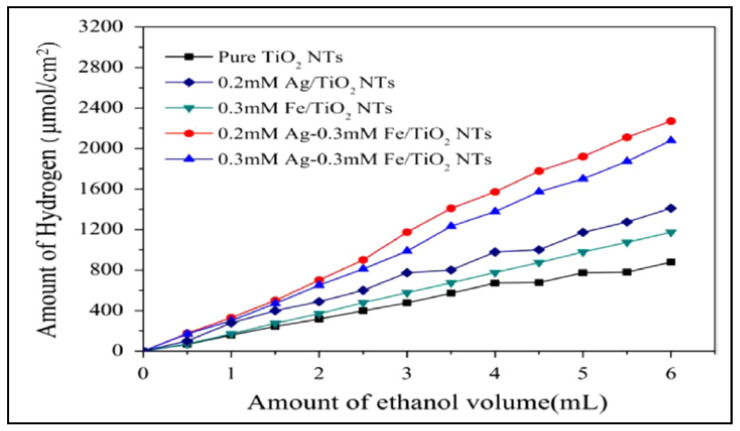
Hydrogen production by water splitting over TiO_2_ catalysts. Reproduced with permission from reference [139].

The highest hydrogen production (1.35 µmol cm^−2^ h^−1^) was achieved with the catalyst containing 0.2 mM of Ag and 0.3 mMFe (0.2 mM Ag–0.3 mM Fe/TiO_2_) (see Figure 25).

**Figure 26:**
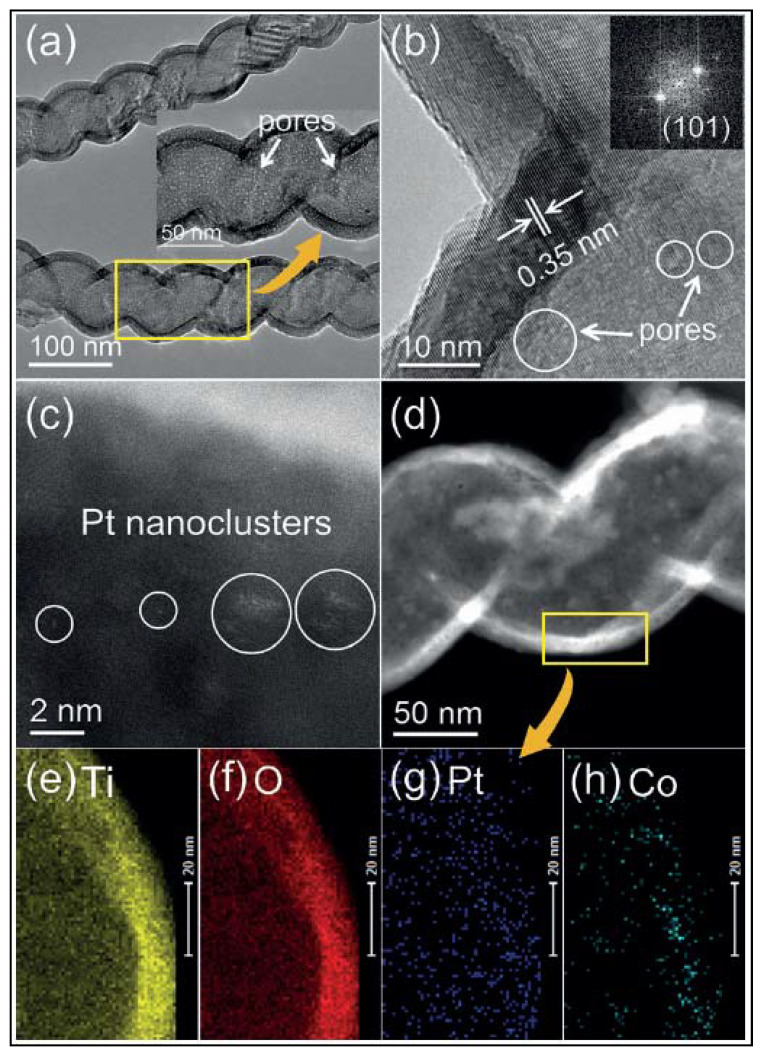
(**a**) TEM and (**b**) HRTEM images of the CoOx/TiO_2_/Pt samples (inset in (**a**), expanded view of the rectangular area; inset in (**b**), fast Fourier transform (FFT) of crystalized TiO_2_ film). (**c**) Atomic-resolution HAADF-STEM image of the CoOx/TiO_2_/Pt samples. (**d**–**h**) STEM image and EDS mapping profiles of the rectangular area in panel (**d**) for (**e**) titanium, (**f**) oxygen, (**g**) platinum, and (**h**) cobalt. One ALD cycle for Pt and CoOx. Reproduced with permission from reference [143].

Atomic layer deposition (ALD) is a method that has also been used to synthesize nanotubes. This process consists in a chemical route for thin film deposition wherein a sequence of self-limiting surface reactions is repeated a discrete number of times [142]. Zhang et al. [143] synthesized porous tubular CoOx/TiO_2_/Pt photocatalysts with spatially separated dual cocatalysts, Pt and CoOx, and measured their catalytic activity by the production of hydrogen in an aqueous methanol solution. Porous TiO_2_ NTs were obtained using carbon nanocoils (CNCs) as sacrificial templates. Figure 26 shows the TEM, HRTEM, Fast Fourier Transform (FFT), HAADF-STEM, STEM and EDS of the different catalysts. Figure 26a,b show the TEM and HRTEM images of CoOx/TiO_2_/Pt structures. TiO_2_ nanotubes are characterized by having uniform wall thickness (ca. 11.4 nm) and uniformly distributed nanopores, with average size of 1.5 nm. The HAADF-STEM (Figure 26c) shows individual Pt atoms as well as Pt nanocluster with dimension of less than 1 nm. The STEM and EDS (Figure 26d–h) analysis confirmed that Co was distributed on the outer surface of TiO_2_ nanotubes. The highest hydrogen production measured with these catalysts was nearly five times higher than those observed with pristine nanotubes (275.9 µmol h^−1^).

Nanofibers have also been used to produce hydrogen via water splitting. The relevance of nanofibers is based on the many basic components that constitute them, and infinite combinations can be synthesized depending on the applications to which the material is going to be dedicated [144].

**Figure 27:**
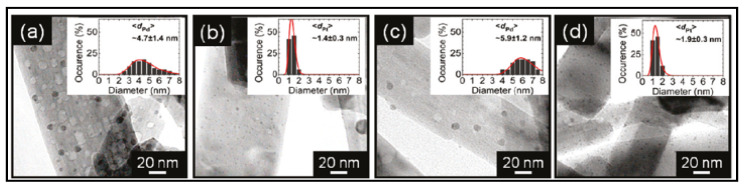
TEM images of (**a**) N-TiO_2_(A)-Pd NF, (**b**) N-TiO_2_(A)-Pt NF, (**c**) N-TiO_2_(B)-Pd NF, and (**d**) N-TiO_2_(B)-Pt NF. In each case, metal loading is ~1.0 wt.%. Insets show histograms of the corresponding metal nanoparticle size distribution. Reproduced with permission from reference [145].

Nanofibers can be obtained by a hydrothermal method. Wu et al. [145] reported the synthesis of N-doped TiO_2_ nanofibers. By doping TiO_2_ with nitrogen, the bandgap of the n-type TiO_2_ could decrease due to the mixing of N 2p states with O 2p states. Additionally, Pt and Pd nanoparticles were incorporated to the as-synthesized N-TiO_2_ nanofibers by a wet impregnation process. The TEM images and the nanoparticle size distribution of the different catalysts are shown in Figure 27. The metal nanoparticles were well dispersed on the surface, and the average size of Pt NPs was considerably smaller than that measured for Pd on both types of supporting surfaces. 

The hydrogen production of the N-doped samples was higher than their undoped counterparts, showing a good efficiency at the two wavelengths analyzed.

**Figure 28:**
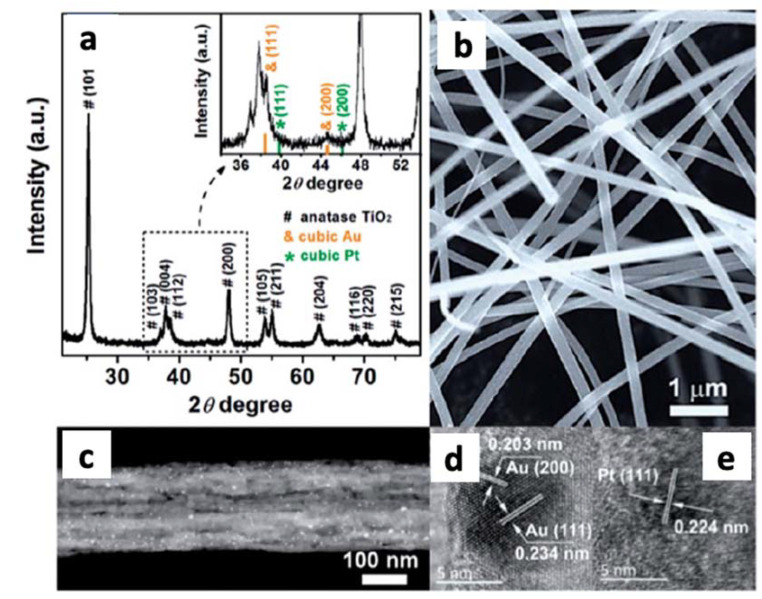
(**a**) XRD pattern, (**b**) SEM image, and (**c**) dark-field STEM image of the Au_0.75_/Pt_0.25_/TiO_2_ nanofibers; HRTEM imafes of (**d**) Au NPs. Reproduced with permission from reference [146].

**Figure 29:**
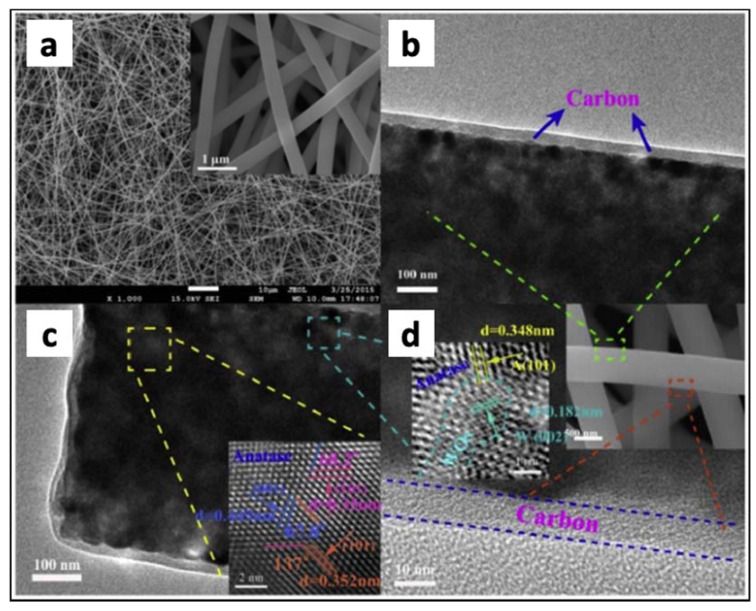
(**a**) FESEM image of the 1% carbon coated TiO_2_/WO_3_ nanofibers. (inset: enlarged image of Figure a); (**b**–**d**) HRTEM images of 1% carbon coated TiO_2_/WO_3_ nanofibers. Inset c: HRTEM of the TiO_2_ area indicated by yellow imaginary line frame; inset d: HRTEM of the WO_3_ area indicated by blue imaginary line frame. Both c and d proved that the nanofiber was coated by a thin carbon layer. Reproduced with permission from reference [148].

Electrospinning is another method to synthesize nanofibers [146]. In fact, nanofibers of very varied composition have been obtained, including carbon nanofibers with different metals, metal oxides or more complex structures, with interesting applications in the catalytic production of hydrogen [147]. TiO_2_ nanofibers were obtained by this approach, and subsequently decorated with Au and Pt nanoparticles to study the plasmon enhancement on the photocatalytic hydrogen production. The XRD patterns, SEM, dark-field STEM and HRTEM images of the Au_0.75_/Pt_0.25_/TiO_2_ nanofibers are shown in Figure 28. The XRD pattern (Figure 28a) shows the signals of anatase TiO_2_ and the cubic phase of Au, but Pt was not detected, probably due to the low concentration. The nanofibers (see Figure 29b) have an average diameter of ca. 190 nm with lengths up to several micrometers. The dark field STEM (Figure 29c) indicates that the metal NPs were deposited through the nanofiber, with an average size of 7.2 nm. The HRTEM images (Figure 29d) show the interplanar distances of 0.234, 0.203, and 0.224 nm, corresponding to the lattice spacing of the Au (111), Au (200), and Pt (111) planes, respectively.

The hydrogen production was measured using a dual beam irradiation of 420 nm and 550 nm. The results showed that the Au/Pt/TiO_2_ nanofibers exhibited certain activity for H_2_ generation under single irradiation at 420 nm that excites the defect/impurity states of TiO_2_. When secondary irradiation at 550 nm was introduced to simultaneously excite Au surface plasmon resonance, higher activity for H_2_ generation was observed.

Hu et al. [148] also employed an electrospinning method to synthesize TiO_2_/WO_3_ nanofibers. WO_3_ is a semiconductor with a narrow band gap (~2.7 eV) and suitable band edges which can match well with TiO_2_ to form a direct-solid-state Z scheme system. This would allow the CB of TiO_2_ to act as strong reducing agent whereas the VB of WO_3_ would exhibit strong oxidizing properties. Additionally, TiO_2_/WO_3_ nanofibers were coated with carbon as sensitizers for increasing the absorptivity at wavelengths ranging from 400 to 800 nm. The SEM and HRTEM images of the 1% carbon coated TiO_2_/WO_3_ nanofibers are shown in Figure 29. The 3D network structure is composed of uniform and straight nanofibers (see Figure 29a). TEM images of Figure 29 show that the thickness of the carbon layer is about 10 nm, coating the TiO_2_ core. The inset in Figure 29c shows three lattice spacings (0.352 nm, 0.35 nm, and 0.445 nm) corresponding to the (101), (−101), and (001) planes of anatase TiO_2_. Another set of the fringes spacing ca. 0.182 nm were ascribed to the (002) lattice spacing of tungsten trioxide, which was dispersive in TiO_2_ matrix.

The hydrogen production with these catalysts was enhanced compared with pure TiO_2_ nanofibers and TiO_2_/WO_3_ nanofibers. This effect was attributed to the multichannel-improved charge-carrier photosyn-thetic heterojunction system with the carbon layer on the surface of TiO_2_ as an electron collector and WO_3_ as a hole collector, leading to effective charge separation on these components. Furthermore, the addition of WO_3_ promoted the graphitization of the carbon layer, improving the transport of electrons in the composite.

### 3.2. Piezoelectric and Thermoelectric Materials

Piezoelectric effect is the ability of some materials to produce an electrical charge in response to applied mechanical stress. This effect is reversible, and also includes the opposite behavior, that is, the generation of mechanical stress when an electric field is applied to the material. Since the first nanomaterials capable of showing this effect were reported, various high-performance materials have been developed with interesting applications from an energy point of view. ZnO nanowires (ZnO NWs) are characterized by a hexagonal structure with significant anisotropy along the c axis, and perpendicular to it, so the application of stresses on this material gives rise to a piezoelectric effect [149,150,151]. When the curvature of the material is caused, a displacement of the cations and anions that form the nanowire structure takes place, which causes the appearance of a dipole that, macroscopically, will cause the appearance of an electrical potential.

In general, this effect can be observed in certain nanowires and nanobelts because, in this conformation, the materials can withstand great mechanical stresses. These materials include those based on ZnO, GaN, InN, CdTe, CdSe, and others, with really high efficiencies for practical purposes (i.e., 0.4V in ZnO [150], 0.35V in GaN [152], 0.3 V in CdTe [153], 60 mV in InN [154], or 137 mV in CdSe [155]. Of these materials, ZnO is by far the easiest to obtain; it is eco-friendly with the environment, and the synthesis of large quantities can be obtained efficiently and at low temperature [150]. Other materials with large piezoelectric coefficients include some ferroelectric nanowires such as Pb(Zr,Ti)O_3_ [156], and BaTiO_3_ [157]. Xu et al. [158] reported high output voltages for Pb(Zr,Ti)O_3_, with values as high as 0.7 V. In the case of BaTiO_3_ nanotubes, with perovskite structure, output voltages of up to 5.5 V have been obtained, under a stress of 1 MPa [159]. When this material is synthesized in the form of thin films by rf magnetron, the output voltages are certainly lower, with values that can reach 1V. Other interesting materials capable of presenting a high piezoelectric response are represented by composites. One of them is the NaNbO_3_ nanowire PDMS polymer composite, with which up to 3.2 V has been obtained. Of all the materials described so far, vertically aligned Pb(Zr_0.52_Ti_0.48_)O_3_ nanowires with an output voltage of 209 V are one of the most efficient systems.

In contrast to the piezoelectric materials described above, capable of generating a voltage when subjected to mechanical stresses, there are some materials capable of converting temperature differences to electricity and vice versa. If we consider that the vast majority of energy consumption processes waste more than half of this in the form of heat, there is no doubt that having systems capable of transforming this heat into reusable energy would be very advantageous. Thermoelectricity is based on the Seebeck-effect, and is due to the different Fermi electron distribution as a function of temperature. Although this effect was initially observed in bimetal junctions, thermoelectric materials are now generally based on semiconductor alloys of Co, Bi, Te, Pb, or Sr. The process implies that a temperature difference occurs between the connected ends of p-type and n-type semiconductors, causing the free carriers to diffuse from the hot side to the cold side, generating a potential difference between both ends. Traditionally, 1D materials capable of exhibiting this effect have been dominated by bismuth. This semimetal, when found with low dimensionality, as in the case of nanowires, is characterized by a band structure and an appropriate electron distribution to show these effects [160].

The basic property of the material that governs the efficiency of thermoelectric generators is the Figure of thermoelectric merit, defined as Z = S^2^σ / κ, where S is the Seebeck coefficient, or thermoelectric power, and σ and κ are the electrical and thermal conductivity, respectively [161]. Z is generally multiplied by the average temperature T to produce a number ZT, which is the parameter used to determine the efficiency of the material. The most advanced thermoelectric materials show a ZT > 3. In order to achieve this, the material is required to have high electrical conductivity (σ), and low thermal conductivity (κ), which is not obvious. One way to achieve materials with this double behavior is through the use of 1D-composites [162]. In this sense, 1D organic composites have recently been developed with significant improvements. Among these, we can mention poly(3,4-ethylenedioxythiophene): p-toluenesulfonic acid (PEDOT: p-TSA), which is synthesized on glass fiber. In this material, and after post-processing, S and especially σ experienced a significant increase, with a substantial improvement in behavior [163]. Other nanostructured organic materials based on carbon nanotubes have shown power factors (PF) of up to 95 [164]. Materials based on PbTe-modified PEDOT nanotubes have also shown high values of S, although in these cases the electrical conductivity is low [165]. Perhaps, future developments of thermoelectric materials will mainly include conductive polymers, whose doping will make it possible to control impurities and defects in the material, allowing to effectively regulate the carrier mobility.

### 3.3. Electrochemical Energy Storage

Electrochemical energy storage devices (EESDs) have significantly increased their presence in our day to day over the last few decades. They have allowed the development of many portable electronics, and as they evolve, new applications arouse. Lately, the interest in electric vehicles has accelerated the interest in EESDs, and their practical applications now range from small and flexible wearables to large grid level systems. Despite the huge improvements over the last few decades, there is a constant strive to improve the energy/power density of the EESDs as new and more complex application are developed.

In this section, we will focus in two types of EESDs, the metal ion batteries, with special focus on lithium ion, and the supercapacitors.

#### 3.3.1. Batteries

Among the battery systems available today, rechargeable lithium ion batteries (LIBs) are the most common and the ones with higher commercial importance due to their outstanding energy density. However, state-of art LIBs are approaching their energy density boundary and new materials and structures are being developed to push this boundary further and meet the ever-increasing energy storage demand.

**Figure 30:**
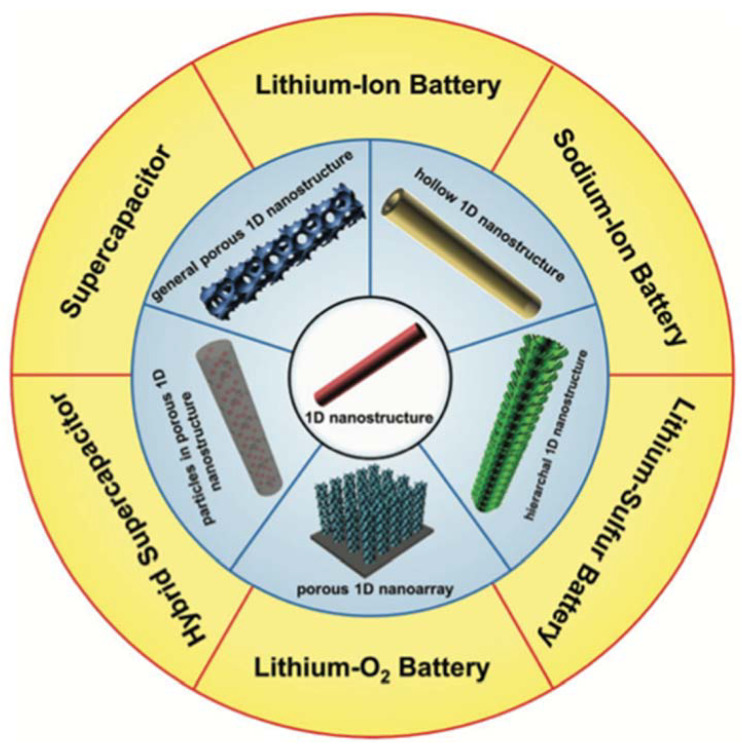
Porous 1D nanostructures and potential applications in electrochemical energy storage. Reprinted with permission from reference [166].

Batteries are usually characterized by high energy density but mediocre power density. Their limitations come from the energy storage mechanism, which is based on redox reactions that takes place in the volume of the electrode material. The incorporation of the metal ions into the bulk of the material requires the diffusion of the latter from the electrolyte to the reaction site, which is a process usually slow. This is the root of the low power density and there is currently a great effort being made to improve it. In this aspect, nanomaterials, and specifically 1D nanomaterials, are a big asset. Their high surface to volume ratio reduces the diffusion distances while their high aspect ratio assures good long-range conduction, dramatically improving their charge/discharge rates [166,167,168] (see Figure 30).

Another important issue for the batteries is cycle performance. High capacity materials tend to be mechanically unstable upon cycling because of the expansion and shrinking produced during the accommodation of the metal ions. This mechanical stress induces the pulverization of the active material which impacts the battery life by the loss of contact of the crumbled pieces. In this regard, the nano scale can also help to improve the stability of the materials, reducing the degradation by buffering the size changes and therefore increasing the lifetime of the devices [169,170].

A number of other benefits can also be ascribed to the 1D nanomaterials in LIBs, such as good flexibility compared to 2D and 3D nanomaterials [171,172,173], the capability to create porous or hollow structures [166], or the possibility to create more complex structures that can easily be grown on thin films to form flexible, self-standing energy storage devices [173,174].

One dimensional materials can be present in the LIBs fulfilling two different functions: as an active material or as a conductive material. The advantages and representative examples of 1D materials in both functionalities in LIBs are summarized in the following points.

##### One Dimensional Active Material

One dimensional nanostructures have recently received a significant attention in respect of their application in batteries. The advantages above mentioned have contributed to the development of an extensive variety of nanostructures (nanorods, nanowires, nanotubes, etc) for even a wider range of materials. Table 1 gives a brief outlook of the variety and diversity of the materials and structures demonstrated in the literature.

In addition to the material and the shape it is presented, the electrode fabrication has also a very decisive importance in nanomaterials. Some of the most attractive properties of the 1D materials are only fully exploited in certain electrode configurations. In particular, the growth of aligned 1D nanostructures on conductive substrates, maximize the exposed surface, providing an efficient electron transfer, deep electrolyte penetration, and good strain accommodation [175,176].

On the other hand, a wide variety of 1D nanomaterials have been developed as active material in LIBs electrodes as a component of the slurry paste (in combination with conductive additives and binders), or fabricating freestanding electrodes. In this case, the key to achieve good electrochemical performances is usually related with the proper arrangement of the materials inside the electrode and the smart combination with other synergetic nanomaterials [52,177].

The active materials for LIBs can be divided into three main groups based on their reaction mechanisms: (1) intercalation, (2) alloying, and (3) conversion. In all of them, 1D materials have been used and a clear performance improvement was accomplished.

- Intercalation

Intercalation is the most common of the lithiation processes in batteries. During this process metal ions are inserted in the outer of the layered materials structure, producing minimal structural changes and therefore provides a stable cycling performance [178]. In opposition to their stability, their capacity is generally low which handicaps their energy density. Carbon materials, titanium dioxide and spinel lithium titanate (Li_4_Ti_5_O_12_, LTO) are the most representative anode materials based on this mechanism. Among the carbon materials, carbon nanotubes (CNTs) have gained huge interest due to the unique structural, electrical, mechanical and electronic properties. In CNTs, Li^+^ has double space to incorporate (inner and outer surfaces) and its flexible morphology offers a stable capacity without pulverization in the electrode [179].

The 1D morphology of nanowires is particularly beneficial to maintaining firm electronic contacts with the conductive agents during charge/discharge cycles. Thus, TiO_2_-based nanowires, nanorods, nanotubes and nanofibers [180,181,182,183,184] have been fabricated, exhibiting excellent high-rate cycling performance.

LTO is a highly appealing anode materials for LIBs due to its extraordinary cycling performance and high safety. Yet, its low conductivity and moderate Li^+^ diffusion coefficient limits its rate capability, and its capacity is even lower than that of the graphite. Still, the 1D nano-structural LTO (a nanorod material (NT-LTO/C) formed by a molecular self-assembly has proven to be a good strategy to improve the properties of the material, shortening the transport lengths, and thereby improving the rate performance [185] of nanorod material (NT-LTO/C) by a novel in situ molecular self-assembly strategy.

- Alloying

Some materials can electrochemically form Lithium alloys in a reversible way. These alloying materials are characterized by high specific capacities and safe operating potentials. While the specific capacity of the alloy based anodes like Si (4200 mAh g^−1^), Ge (1600 mAh g^−1^), Sn (994 mAh g^−1^), etc., are more than graphite (372 mAh g^−1^), the poor cycling stability and the irreversible capacities at the initial cycles limit their practical applications [186,187]. These effects arise from the swelling/shrinking during lithiation/de-lithiation, reaching volume changes up to 400%, which results in pulverization of the active materials and lose of electrical contact. To overcome these inherent limitations, it has been proven that 1D nanostructures help to release the stress without breaking which helps to retain the capacity [188,189].

A wide selection of 1D nanomaterials have been used as LIBs alloy anodes [190,191] and comparatively, their electrochemical performance has been shown to be significantly improved compared to the same material in different morphologies. Some examples are displayed in Table 1.

- Conversion

At the turn of the 21st century, new perspectives for the development of LIBs brought interest in the search of a new concept of reactivity with Li, different from those of intercalation and alloy with Li. These circumstances encouraged the investigation of materials with new functional mechanisms; those can make the reactions of "conversion" with lithium. The reversible electrochemical reaction of lithium with transition metal oxides or sulfides, conventionally called the "conversion reaction" [192].

Through this multi-electron transfer process, conversion-type materials can easily accommodate more Li ions to achieve high specific capacities. Conversion type materials such as transition metal oxides (TMOs) have become a promising alternative to graphite due to their safety, low cost and the high theoretical specific capacity. However, the use of these conversion materials also has its drawbacks, such as low conductivity, low initial coulomb efficiency, instability during long cycling, and high-volume expansion, which limit their application in LIBs. Some of these limitations that can be overcome using nanostructures, such as 1D metal nanostructure arrays oxides, sulphides, and hybrid structures, as shown in Table 1.

##### One Dimensional Conductive Agent

One of the most common drawbacks of nanomaterials is their low conductivity and poor connection with the conductive network composing the electrode [193,194]. Regarding these limitations, an approach that has become popular lately is the use of carbon nanotubes (CNTs) and nanofibers (CNFs) [195,196]. As opposition to other conductive agents, lD conductive materials keeps long range of interconnection of active material particles, while maintaining high porosity and allowing the electrolyte to penetrate deeper into the electrode.

The 1D carbon nanostructures cannot only provide better electrical connection to the active materials, but also their porous structures are beneficial allowing the accommodation of the volume expansion [179,195]. Furthermore, 1D carbon nanomaterials provide good mechanical robustness and flexibility to the electrodes due to their excellent mechanical properties. 

In addition, the good interconnection that they provide, it allows a much lower weight than other additives, further enhancing the energy density of the electrodes. This approach is quite mature, and it has become a standard for the battery manufacturers, being currently applied by OCSiAl (carbon nanotube manufacturer) in partnership with Shenzhen BAK Power Battery (China), Haiyi Enterprise (China), and Polaris Battery Labs (USA).

#### 3.3.2. Supercapacitors

In opposition to batteries, supercapacitors, have a very high-power density with much lower energy density. The energy storage mechanism is based on electrostatic charge accumulation on the surface of the electrode materials (electric double-layer capacitors, EDCLs) or on fast reversible redox reactions on the surface of the materials (pseudocapacitors, PCs). The raw capacitance of the material usually depends on the amount of available surface, being one of the reasons why the nanomaterials have attracted so much attention in this field. It is also worthy to highlight the importance of the porosity and conductivity of the materials that compose the electrodes. These two properties are extremely important to keep the high-power density that characterizes the supercapacitors, and in this regard, 1D nanomaterials show very promising candidates [164].

##### EDLCs Materials

This type of supercapacitors is characterized by a very fast charge/discharge response due to the on-surface adsorption of ions, usually achieving much higher rates than PC supercapacitors. Additionally, the limited interaction between adsorbed ions and the inner structure of the material grant them long-term stability and longevity. On the other hand, the high current and fast rates involved in the operation of this devices, makes necessary very high conductivities and only low resistive materials can be used both as active materials and as part of the composite.

The advantages that the 1D materials bring to EDLCs, mainly come from the high surface area and long-range material interconnection. Since the charge is built up at the electrode/electrolyte interface, the improvement of the later directly affects the capacitance. Historically, the most common material for EDLCs is carbon as it compromises high conductivity, high stability (both chemical and mechanical) and low cost [166]. In the nanoscale, carbon is also the main choice for the EDCLs and the most common shapes in which it is applied are nanofibers [218,219] and nanotubes. Commonly, CNTs electrodes do not show a surface area as high as other carbon materials such as activated carbon [167,220] and to overcome this problem, usually, two approaches are followed: 1) porosity increase by chemically treating the CNTs and 2) vertically align the CNTs to allow deeper electrolyte penetration.

The first approach is further discussed in the pseudocapacitance section, as this type of treatments, aside of increasing the surface, usually adds functional groups that provides pseudocapacitive behaviour. Still, pure EDLCs made from porous CNTs can be found. For instance, Xu et al. [220] were able to increase CNTs capacitance from 18 F/g to 54 F/g after KOH treatment. More common is to find the use of aligned CNTs to enhance the electrolyte penetration, which has proved to provide good performance [221,222] and feasible in roll to roll synthesis [223]. More examples of these two types can be found in Table 2.

##### Pseudocapacitors

By definition, pseudocapacitive materials should have a linear dependence of the charge with the potential window (capacitance should be constant over a voltage window) [224]. However is well accepted that systems with redox reversible peaks with no separation and without phase changes, can be also considered pseudocapacitive materials [225].

Despite the very similar macroscopic response from the EDCLs and pseudocapacitive materials, the nature of their capacitance is clearly different, being non faradaic for the first and faradaic for the later. Pseudocapacitive materials are characterized by a much higher capacitance than the EDLCs, but due to the kinetics of the redox reactions, their charge/discharge rates are usually slower. It is important to highlight that there is currently a huge confusion and discussion in the literature with the proper classification of some pseudocapacitive materials. Specially with TMOs, many authors have miss labelled classic battery-like behaviours with pseudo-capacitive ones. Example of this are Ni(OH)_2_, Zn(OH)_2_, Co_3_O_4_, IrO_2_ or NiCo_2_S_4_ as discussed in references [224,226]. All of these materials show distinctive redox peaks that makes the use of capacitance as charge storage metric, simply wrong. The miss-use of capacitance, has deeper implications than just wrong categorization, and can lead to a huge inaccuracy when calculating the energy density, as illustrated in reference [225]. The correct classification of pseudocapacitive materials becomes even more confusing when the size of the materials goes below a certain size threshold. Due to the short diffusion distances, the ions insertion/extraction in the nanosized battery-type materials are much faster (because of the time scale) and their voltagrams show linear relationships between voltage and time, not displaying the common redox plateau and showing a capacitive-like behaviour [227]. This is named by some authors “extrinsic” pseudocapacitance [228] but as discussed in the references [224,226,228], it should not be confused with true pseudocapacitive materials due to the differences in their performance and on the reaction kinetics.

Taking this discussion into account, in this section, we will only consider true pseudocapacitive materials. However, it is worth noting that the use of battery-type nanomaterials in combination with capacitive electrodes to assemble hybrid energy storage devices (HESDs), is a valid strategy that lately is getting more attention and interest [229]. Those devices won’t be reviewed here as they will require a more extensive discussion, but more information can be found in literature [229,230].

The most common pseudo-capacitive materials are: heteroatom-doped carbonaceous materials, conductive polymers and some transition metal oxides (RuO_2_, MnO_2_, V_2_O_5_). Similar to EDLCs, the nanostructuration of pseudocapacitive materials helps to bring the material/electrolyte interface up, increasing the number of accessible active redox sites. Even in bulk, the whole mass of the pseudocapacitive materials is theoretically accessible for charge storage through ion diffusion. However, the fast operation of the SCs only allows the most superficial material to contribute to the capacitance. This is where the nanomaterials really shine, as they can potentially make the whole mass accessible for energy storage at very fast operation rates.

In comparison with common EDLCs, one of the problems of pseudocapacitive materials is their conductivity. Most of them have poor conductivity that, due to the very high operation current densities, the polarization coming from the electrode resistance have a huge impact on the device output. This can be overcome using conductive additives, commonly, carbonaceous species as discussed in the battery section.

- Doped Carbonaceous Materials

A common approach to increase the performance of carbonaceous materials in pseudocapacitors (SCs) is their chemical activation and functionalization. These processes introduce defects and functional groups in the materials, enabling a pseudocapacitive behaviour that enhances the specific capacitance of the materials [226]. As a side effect, the functionalization processes are usually accompanied by a substantial porosity increase but also a change on the resistance and self-discharge characteristics [231]. There are many types of different functional groups which influence the energy storage properties differently. For instance, oxygen and nitrogen groups increase carbon nanostructures capacitances [232], while carboxyl groups improve the hydrophilicity in aqueous electrolytes [231]. Some examples can be seen in Table 2, where one can observe a significant performance increase compared to EDLCs materials.

- Conductive Polymers

They are cheap, easy to synthetize, have pseudocapacitive behaviour in the whole volume, high capacitance (PANI 1284 F/g, PPy 480 F/g and PEDOT 210 F/g [233]) and good conductivity [226]. However, they are accompanied by a few drawbacks too: they swell and shrink during charge/discharge affecting the mechanical integrity of the electrodes [234], they show poor ion mobility [235] and they have a reduced working potential range [233]. Combined, these effects usually produce poor cycling stability [233] which prevent any commercial application. Designing 1D nanostructures from these conductive polymers can effectively supress some of their drawbacks in a similar way as described for alloying materials in the LIBs section. Moreover, as in the CNTs, it is a common practice to use vertically aligned nanowires to ensure the electrolyte infiltration and a fast ion exchange [236] (Table 2). Due to its higher capacitance, PANI is the most common conductive polymer to use as standalone material (Table 2), while PPy and PEDOT are commonly used as conductive/capacitive additive with other capacitive materials [237,238,239].

- TMOs

The first pseudocapacitive material studied was a TMO, specifically, the RuO_2_. Actually, the definition of pseudocapacitance was introduced by Conway et al. [239] while studying the RuO_2_. The nanostructuration of RuO_2_ has proved to maximize the exposed surface and therefore its performance, obtaining capacitances over 1000 F/g at decent rates [240]. However, RuO_2_ price and availability restrict its application, and cheaper alternatives has been actively explored. One of the most promising ones is MnO_2_, which have a lower cost and high theoretical capacitance (1100–1300 F/g). Nevertheless, only a very thin superficial layer of the material is electrochemically active [226], so it usually shows lower capacitances than RuO_2_. For this reason, the preparation of MnO_2_ nanostructures has been such a common approach for SCs, as it helps to improve the material utilization. A few examples can be found in Table 2. It is also important to highlight that MnO_2_ does not have any oxidation states below 0 V, what limits its use in symmetric devices [234].

There are other well studied pseudocapacitive materials such as V_2_O_5_ and other similar layered materials (such as MoO_3_, Nb_2_O_5_ or H_x_Ti_y_O_x_) that shows what is known as intercalation pseudocapacitance. In this type of pseudocapacitance, the ions diffuse through the layered structure of the materials but their crystallographic structure is not significantly altered and the voltametric response is not diffusion limited, which distinguishes it from the intercalation in batteries [225,226].

## 4. Summary and Outlook

Current technological advances and developments require the use of reliable sources of energy that guarantee the sustainability of our near future. Over the last 30 years, increasingly evolved systems have been developed that allow the best use of the planet’s energy resources. Solar cells, piezo, and thermoelectric generators, and even obtaining hydrogen as an energy vector, are examples of an unprecedented development towards a more technologically advanced and sustainable world. The developments in increasingly efficient energy storage systems, specifically batteries and capacitors, already allow energy autonomy that is crucial for the vast majority of devices to which we are accustomed. For all of these applications, and others that will emerge over the years, 1D nanostructured materials have shown promising prospects for improving efficiency and will be key in new developments that promote a sustainable future for humanity.

## Figures and Tables

**Table 1:** Examples of different 1D battery nanomaterials by structure and storage mechanism.

Nanorods	Nanowires	Nanotubes	Nanocables
^3^ ZnMnO_3_ [197]	^2^ Si [170]	^1^ g-CNTs [179]	^2^ Cu-Si [198]
950 mAh/g (0.5 A/g) 500 cycles	1200 mAh/g (2 A/g) 500 cycles	200 mAh/g (0.5 A/g) 400 cycles	1500 mAh/g (1.4 A/g) 100 cycles
^3^ ZnCo_2_O_4_ [199]	^2^ Si [200]	^3^ Co_3_O_4_ [201]	^1,2^ SnO_2_-TiO_2_ [202]
1050 mAh/g (0.4 A/g) 200 cycles	900 mAh/g (0.2 C) 100 cycles	1800 mAh/g (0.3 A/g) 100 cycles	300 mAh/g (0.1 C) 50 cycles
^2^ β-Sn [203]	^1^ TiO_2_ [52]	^3^ ZnMn_2_O_4_ [204]	^3^ CNT@Fe_3_O_4_@C [205]
600 mAh/g (0.2 C) 100 cycles	350 mAh/g (0.02 A/g) 35 cycles	670 mAh/g (0.2 A/g) 280 cycles	700 mAh/g (2 A/g) 200 cycles
^3^ α-Fe_2_O_3_ [206]	^2^ Ge [207]	^2,3^ SnO_2_-CuO [208]	^1^ MWNT@LTO [209]
970 mAh/g (0.5 C) 100 cycles	900 mAh/g (0.5 C) 1100 cycles	600 mAh/g (0.5 A/g) 100 cycles	130 mAh/g (10 C) 100 cycles
^3^ CuO [210]	^2,3^ Zn_2_GeO_4_ [211]	^2^ Si [212]	^2^ Ni-Si [213]
670 mAh/g (0.1 A/g) 150 cycles	1200 mAh/g (0.1 C) 100 cycles	600 mAh/g (12 C) 6000 cycles	1100 mAh/g (0.5 C) 100 cycles
^3^ V_2_O_3_ [214]	^3^ WO_3_ [215]	^3^ Zn_4_Sb_3_ [216]	^2,3^ Ag@γ-Fe_2_O_3_ [217]
200 mAh/g (0.1 C) 125 cycles	660 mAh/g (0.28 C) 140 cycles	450 mAh/g (0.1 A/g) 100 cycles	890 mAh/g (0.1 C) 60 cycles

^1^ Intercalation material; ^2^ Alloying material; ^3^ Conversion material.

**Table 2:** Summary of supercapacitors performance for different active materials.

Storage Mechanism	Active Material	Electrode Composition	Capacitance (F/g)	P–E *	Ref.
EDLCs	Carbon	MWCNTs/CB/PVDF (85/5/10)	135 F/g (1 mV/s)	-	[241]
EDLCs	Carbon	Single-wall CNTs	150 F/g	20 k W/Kg 6.5 Wh/kg	[242]
EDLCs	Carbon	Carbon nanofibers + CNTs	130 F/g (5 mV/s)		[243]
EDLCs	Carbon	Vertically Aligned CNTs + CNFs	180 F/g (150 A/g)	40 kW/Kg 20 Wh/Kg	[244]
PS	Functionalized Carbon	Oxygen functionalized CNT fibres	46 F/g (50mV/s)	20 kW/Kg 1.29 Wh/Kg	[245]
PS	Functionalized Carbon	Vertically aligned, Oxygen functionalized CNTs	440 F/g	100 kW/Kg 100 Wh/Kg	[246]
PS	Functionalized Carbon	Template based, vertically aligned CNTs	365 F/g (2 A/g)	-	[247]
PS	Functionalized Carbon	N–doped CNF network	175 F/g (50 A/g)	1200 W/Kg 5.9 Wh/Kg	[248]
PS	Functionalized Carbon	N–doped CNTs	228 F/g (1 mA/cm^2^)	7.75 kW/Kg 29 Wh/Kg	[249]
PS	TMO–RuO_2_	Hydrous RuO_2_ nanotubular array	≈1000 F/g (100 mV/s)	4320 kW/Kg 7.5 Wh/Kg	[250]
PS	TMO–MnO_2_	MnO_2_ nanotube array	325 F/g (2 A/g)	-	[251]
PS	TMO–MnO_2_	MnO_2_ NW (80%)/CB (15%)/PTFE (5%)	279 F/g (1 A/g)	-	[252]
PS	TMO–V_2_O_5_	V_2_O_5_ nanowires/CNTs	216 F/g (5 mV/s)–460 F/cm^2^	6.5 kW/L 29 Wh/L	[173]
PS	TMO–V_2_O_5_	V_2_O_5_ + PPy	308 F/g (0.1 A/g)	2.5 KW/Kg 24 Wh/kg	[253]
PS	Conductive polymer	PPy nanowires arrays	250 F/g (2.75 A/g)	10 kW/Kg 50 Wh/Kg	[236]
PS	Conductive polymer	PPy nanowire network	332 F/g (1 mA/cm^2^)	7.75 kW/Kg 29 Wh/Kg	[249]
PS	Conductive polymer	PANI nanowire hydrogel	636 F/g (2 A/g)	-	[254]
PS	Conductive polymer	RGO–PANI nanowires paper PANI nw/RGO/PANI nw sandwich	956 F/g (1 A/g)–172 F/cm^3^ 363 F/g (1 A/g)–722 F/cm^3^		[255]
PS	Conductive polymer	PANI arrays/graphene foams	936 F/g (1 A/g)	103 kW/Kg 21 Wh/Kg	[256]

* Power and energy density of the reported device.

## Data Availability

Information regarding the work performed in this Review is available from the corresponding authors on reasonable request.
